# Igf1r Signaling Is Indispensable for Preimplantation Development and Is Activated via a Novel Function of E-cadherin

**DOI:** 10.1371/journal.pgen.1002609

**Published:** 2012-03-29

**Authors:** Ivan Bedzhov, Ewa Liszewska, Benoît Kanzler, Marc P. Stemmler

**Affiliations:** Department of Molecular Embryology, Max-Planck Institute of Immunobiology and Epigenetics, Freiburg, Germany; The Babraham Institute, United Kingdom

## Abstract

Insulin-like growth factor I receptor (Igf1r) signaling controls proliferation, differentiation, growth, and cell survival in many tissues; and its deregulated activity is involved in tumorigenesis. Although important during fetal growth and postnatal life, a function for the Igf pathway during preimplantation development has not been described. We show that abrogating Igf1r signaling with specific inhibitors blocks trophectoderm formation and compromises embryo survival during murine blastocyst formation. In normal embryos total Igf1r is present throughout the membrane, whereas the activated form is found exclusively at cell contact sites, colocalizing with E-cadherin. Using genetic domain switching, we show a requirement for E-cadherin to maintain proper activation of Igf1r. Embryos expressing exclusively a cadherin chimera with N-cadherin extracellular and E-cadherin intracellular domains (NcEc) fail to form a trophectoderm and cells die by apoptosis. In contrast, homozygous mutant embryos expressing a reverse-structured chimera (EcNc) show trophectoderm survival and blastocoel cavitation, indicating a crucial and non-substitutable role of the E-cadherin ectodomain for these processes. Strikingly, blastocyst formation can be rescued in homozygous NcEc embryos by restoring Igf1r signaling, which enhances cell survival. Hence, perturbation of E-cadherin extracellular integrity, independent of its cell-adhesion function, blocked Igf1r signaling and induced cell death in the trophectoderm. Our results reveal an important and yet undiscovered function of Igf1r during preimplantation development mediated by a unique physical interaction between Igf1r and E-cadherin indispensable for proper receptor activation and anti-apoptotic signaling. We provide novel insights into how ligand-dependent Igf1r activity is additionally gated to sense developmental potential *in utero* and into a bifunctional role of adhesion molecules in contact formation and signaling.

## Author Summary

One of the most important steps during mammalian development is the formation of a blastocyst before implantation. Proper blastocyst development is fundamentally reliant on the function of the E-cadherin adhesion molecule, which cannot be replaced by another highly related member of the cadherin family. We have addressed the question of how E-cadherin unfolds its unique function during this central embryonic process. We generated mouse mutants that allow specific domain swapping of extra- and intracellular protein domains of E-cadherin with the corresponding portion of N-cadherin. Upon E-cadherin (*Cdh1*) depletion, apoptosis is induced in cells that are required to form the trophectoderm, the outer cells of a functional blastocyst. Uncoupling of the two E-cadherin domains demonstrated that specifically the presence of the extracellular domain is indispensable in providing essential survival cues. To establish a proper trophectoderm the insulin-like growth factor I receptor (Igf1r) is intimately connected to the E-cadherin–mediated suppression of apoptosis. By interaction of the two proteins Igf1r is efficiently activated to allow embryo survival, blastocyst formation, and implantation. This novel and adhesion-independent function of E-cadherin may serve as paradigm for bifunctionality of adhesion molecules and how they are particularly utilized to interpret signal transduction activities in specific cellular contexts.

## Introduction

The ultimate goal of the mammalian preimplantation development is the formation of a hollow shaped embryo called blastocyst, crucial for all stages of subsequent development. It is generated by a highly organized interplay of multiple signaling pathways guiding development from a fertilized egg to an 128-cell staged embryo. At the end of this important process three distinct cell lineages are established – the epiblast, that will give rise to the embryo proper, the primitive endoderm, that forms some of the extraembryonic membranes and the trophectoderm (TE) that contributes to the placenta [1], [2]. In mice, at embryonic day (E)4.5 after segregation of the early lineages is completed the blastocyst hatches from its glycoprotein envelop (*zona pellucida*) in order to invade the uterine epithelium and implant. Besides the orchestrated interplay of transcription factor networks that regulate expression of *Oct4*, *Cdx2*, *Nanog*, *Gata4* and *Gata6*, indispensable for correct lineage segregation [3]–[7], the formation of a proper blastocyst strongly depends on tightly controlled cell adhesion mainly mediated by E-cadherin (E-cad, also known as Cdh1) [8]. Mice deficient for *E-cad* (*Cdh1*) are incapable of forming a proper trophectodermal epithelium [9], [10]. Compaction, however, is accomplished by residual maternally provided gene expression and is lost upon maternal/zygotic *E-cad* (*Cdh1*) depletion [11], [12]. In contrast, N-cadherin (N-cad, also known as Cdh2), another crucial member of classical cadherins is first detected after implantation and its gene ablation demonstrates distinct functions as well. *N-cad* (*Cdh2*) expression is initiated when the first mesoderm cells start to emerge at the primitive streak during gastrulation [8]. Although the mesoderm is properly formed in *N-cad (Cdh2)*-deficient mice, patterning of the somites and the neural tube is severely affected, and embryos die due to a heart defect [13]. Interestingly, the cardiac phenotype is rescued by ectopic expression of *E-cad* (*Cdh1*) in the developing heart, indicating that the adhesive function of cadherins is at least in part interchangeable [14]. In agreement with this finding, E-cad and N-cad have similar properties in the degree of conservation of amino acids (aa), in mediating homophilic adhesion, and in binding to the same intracellular interaction partners, such as β-catenin, Plakoglobin and p120ctn [15], [16]. However, *E-cad* (*Cdh1*) and *N-cad* (*Cdh2*) are usually expressed in a mutually exclusive pattern and induce different cellular properties. Cell polarity and an epithelial sessile shape are established in cells that express *E-cad* (*Cdh1*), whereas cell migration can be induced in cells that gain *N-cad* (*Cdh2*) expression [17], [18]. This phenomenon reflects the contribution of cadherins to epithelial-mesenchymal transition during gastrulation, as well as during carcinogenesis [8], [19], [20]. Detailed knowledge about how the unique properties of the two related cadherins are translated in molecular terms is still limited.

By ectopically switching cadherin expression from E-cad to N-cad using a previously reported gene replacement approach, we were able to maintain cell adhesion and analyze specific and unique molecular features of either E-cad or N-cad [21]. Interestingly, embryos carrying two N-cad knock-in alleles in the *E-cad* (*Cdh1*) locus (N-cad ki/ki) were not able to form a proper TE and died within the *zona pellucida*, similar to *E-cad* ^−/−^ embryos. Since control embryos that harbor an HA-tagged *E-cad* (*Cdh1*) cDNA knock-in allele were able to form blastocysts and implant properly, the result of N-cad ki/ki embryos suggests that E-cad has a unique function during blastocyst formation [21], [22]. However, how this crucial and unique function of E-cad is implemented and why it cannot be replaced by N-cad is a complete enigma.

The insulin-like growth factor I receptor (Igf1r) belongs to the protein family of receptor tyrosine kinases and is mainly activated by Igf1 and Igf2 acting in an autocrine and paracrine manner. The downstream signaling cascade regulates proliferation, differentiation, metabolism and survival of most cell types during fetal growth and postnatal life [23], [24]. Blocking kinase activity by either loss-of-function mutation of the receptor or of both ligands result in reduced body weight and size combined with multiple defects including muscle dystrophy and impaired survival of newborn pups [25], [26]. The Igf1/Igf2/Igf1r axis provides growth promoting, anti-apoptotic functions in almost all tissues and organs and treatment of mouse preimplantation embryos with Igf1 enhanced blastocyst formation *in vitro* by supporting PI3K/Akt activity [27], [28]. However, detailed knowledge about a role of this pathway during preimplantation development is lacking.

Here, we further addressed the question about the unique function of E-cad by replacing its expression with chimeric cadherin genes using similar knock-in approaches as for N-cad ki/+ mice and identified a novel fundamental and cell-adhesion independent function of E-cad in promoting cell survival of the TE by facilitating Igf1r activity.

## Results

### Generation of mice expressing chimeric cadherins under the control of the *E-cad* (*Cdh1*) locus

**Figure 1:**
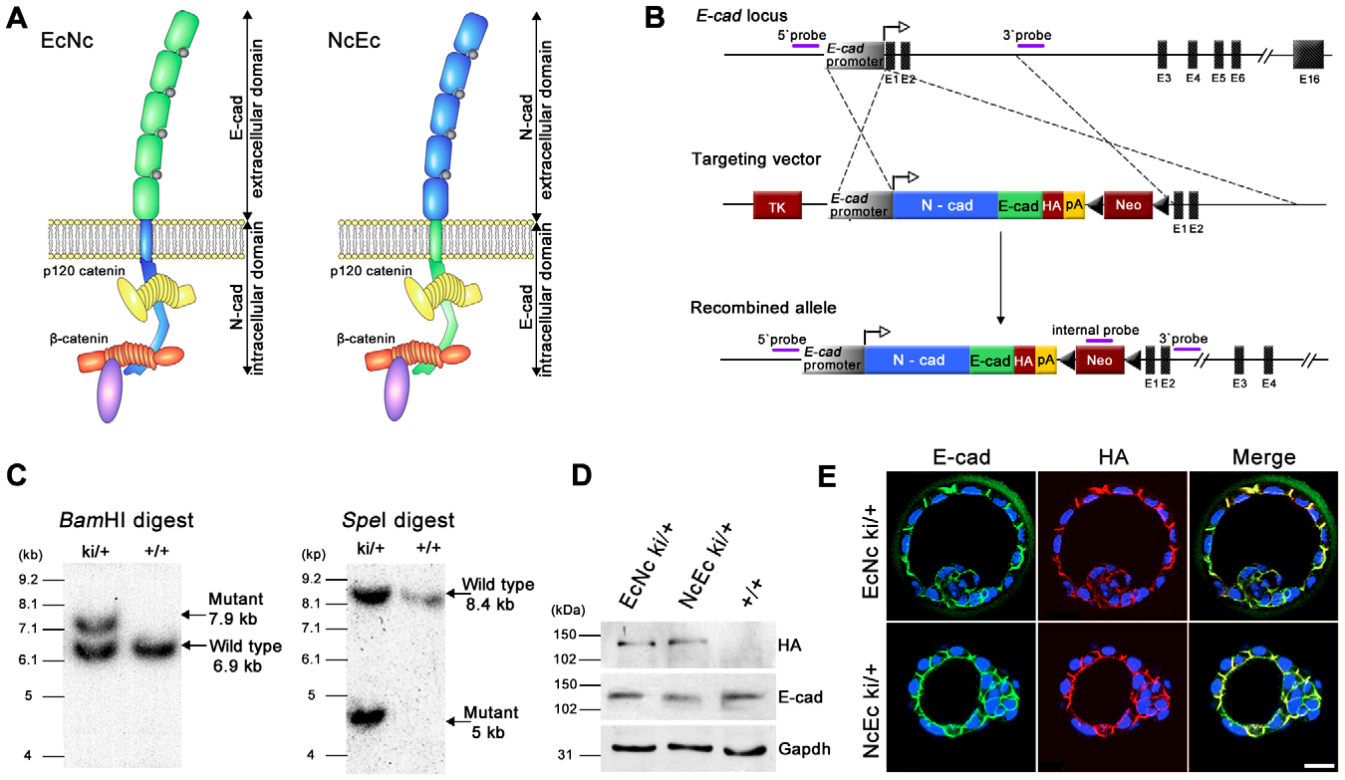
Generation of EcNc and NcEc cadherin proteins expressed in the *E-cad* (*Cdh1*) locus. (A) Schematic representation of EcNc and NcEc protein structure in their cadherin-catenin complex (adapted from [8]). (B) Gene targeting strategy and the resultant knock-in allele, representatively shown for NcEc; TK, *HSV*::*tk* negative selection cassette; HA, haemagglutinin tag; pA, SV40 polyadenylation signal; Neo, neomycin resistance cassette, flanked by loxP sites (black triangles). (C) Southern blot analysis of obtained ES cell clones using the 5′ probe. (D) Expression of the knock-in alleles in ES cells after removal of the neomycin cassette reveals equal expression of both HA-tagged proteins. (E) Immunofluorescence labeling of EcNc (upper) and NcEc (lower panel) showing complete overlap of anti-HA and anti-E-cad staining in heterozygous E3.5 blastocysts in confocal optical sections. Scale bar, 25 µm.

To elucidate the function of E-cad during TE formation, the protein was divided into two parts, its N-terminal extracellular adhesive region and its C-terminal transmembrane and intracellular portion, the latter of which mediates its interaction with catenins. These regions were combined with the matching portions of the N-cad molecule to generate artificial chimeric cadherins (Figure 1A). Cloned cDNAs encoding EcNc (corresponding to aa 1–710 of the E-cad precursor peptide and 725–906 of N-cad) and NcEc (aa 1–724 of N-cad and 711–884 of E-cad) were fused to a sequence encoding an HA-tag and inserted into the *E-cad* (*Cdh1*) locus to replace *E-cad* as described previously (Figure 1B–1D) [21], [22], [29]. For both EcNc and NcEc approaches, two independent ES-cell clones were used to generate the corresponding knock-in strains. Proper expression of the chimeric molecules was confirmed on mRNA level in ES cells and by immunofluorescence and immunohistochemistry of embryos after deletion of the selection cassette. RNA levels of the two knock-in alleles were comparable to the amount of N-cad ki and EcHA transcripts (Figure S1A). Distribution of both chimeric proteins completely overlapped with endogenous E-cad staining in TE and inner cell mass (ICM) cells of heterozygous preimplantation embryos (Figure 1E) and accurately recapitulated *E-cad* (*Cdh1*) expression in the epithelia of post-implantation stages (Figure S1B). The analysis confirmed successful gene replacement and correct spatiotemporal expression of both knock-in alleles.

### Homozygous mutant NcEc embryos fail to form an intact TE layer

**Figure 2:**
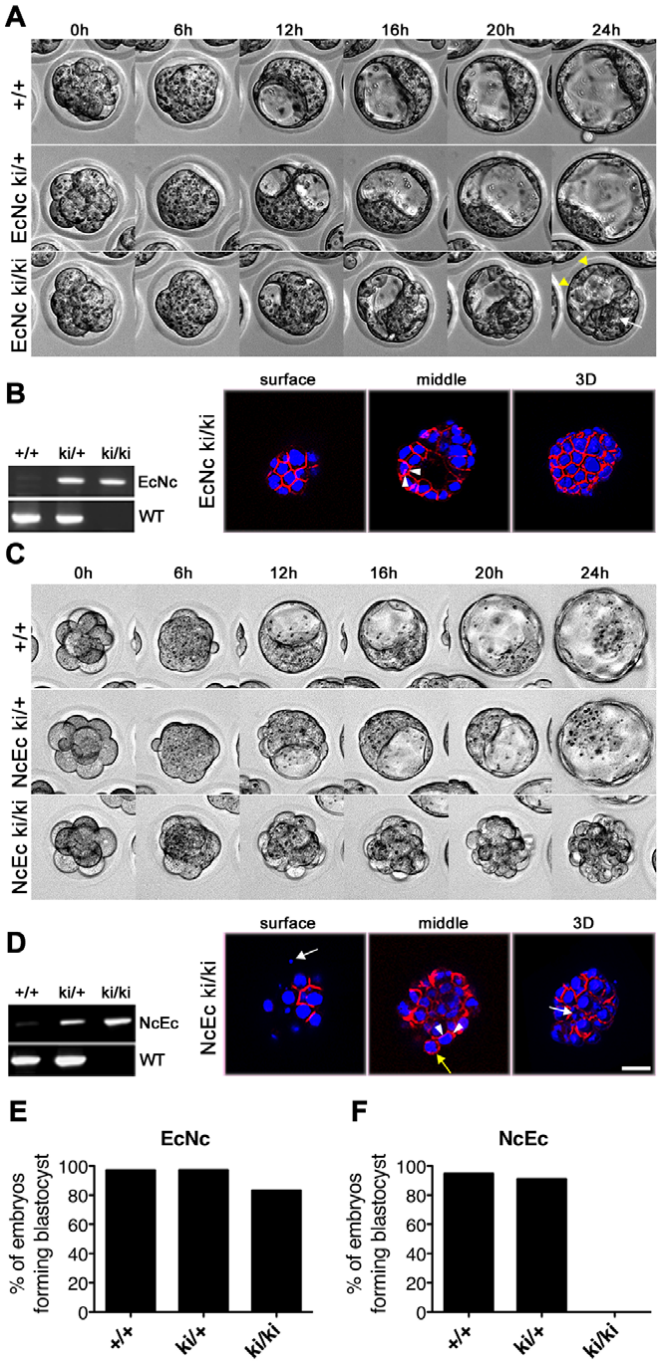
Homozygous NcEc embryos fail to form a functional trophectoderm. (A) Still images from a twenty-four-hour time-lapse recording of *in vitro* cultured embryos from heterozygous EcNc intercrosses at E2.5. All embryos properly compact and separate the ICM (white arrow) from the TE (yellow arrowheads). (B) PCR genotyping of embryos in (A) and HA.11 immunofluorescence labeling of EcNc protein given in surface and medial optical sections, as well as z-stack 3D reconstructions, demonstrating the proper distribution of EcNc at the basolateral membrane (arrowheads). (C) Still images from a twenty-four-hour time-lapse recording of *in vitro* cultured embryos from heterozygous NcEc intercrosses at E2.5. Compaction is accomplished in all genotypes, but NcEc homozygous embryos cannot form a TE layer. (D) Similar analysis of NcEc homozygous embryos as shown in (B). Although NcEc is located mainly at basolateral sites (arrowheads), a uniform distribution was found in several outer cells (yellow arrow), and fragmented nuclei (white arrows) were also detected. (E,F) Percentage of embryos that formed a proper blastocyst in time-lapse experiments and during *in vitro* culture for EcNc (E, n = 33 wt, n = 101 ki/+ and n = 48 ki/ki) and NcEc mutants (F, n = 58 wt, n = 156 ki/+ and n = 56 ki/ki) in >10 independent experiments. Scale bar, 25 µm.

Similar to the N-cad ki/+ mice, no phenotype was detected in heterozygous NcEc or EcNc animals, indicating that the chimeric proteins did not interfere with E-cad-mediated adhesion. At E2.5, EcNc and NcEc homozygous embryos were observed in a 24-h time-lapse experiment to monitor blastocyst formation. Both homozygous mutants underwent compaction normally and were indistinguishable from their heterozygous littermates (Figure 2A and 2C, 0–6 h). EcNc homozygous mutants (EcNc ki/ki), which express the cadherin with the adhesive domain of E-cad, properly segregated the TE from the ICM cells and formed a blastocoel cavity similar to control embryos (Figure 2A, 6–24 h, and 2E). However, the TE was more fragile as pulsing caused by sequential expansion and collapse was observed more frequently in EcNc homozygous mutants than in control littermates (Figure 2A and Video S1). Proper protein localization to the basolateral membranes of TE cells was confirmed by immunofluorescence (Figure 2B). In contrast, homozygous NcEc mutants (NcEc ki/ki), which express the cadherin with the extracellular domain of N-cad, were incapable of establishing a blastocyst. Cells on the outside were shuffled around, rounded up, vacuolated and became scattered at the surface of the embryo, whereas the control littermates formed blastocysts during the 24-h time-lapse recording (Figure 2C, 2F and Video S2). Confocal optical plane sections of immunofluorescently labeled embryos revealed that the NcEc protein was evenly distributed on the scattered cells on the surface (Figure 2D, yellow arrow). The results indicated that TE and blastocoel cavity formation required the presence of the extracellular domain of E-cad. However, the reduced stability of the TE layer in homozygous EcNc mutants showed that the intracellular domain of the E-cad molecule also contributed to the process and has an important function that cannot be performed by N-cad.

### Key features required for blastocyst formation are correctly distributed in homozygous NcEc embryos

**Figure 3:**
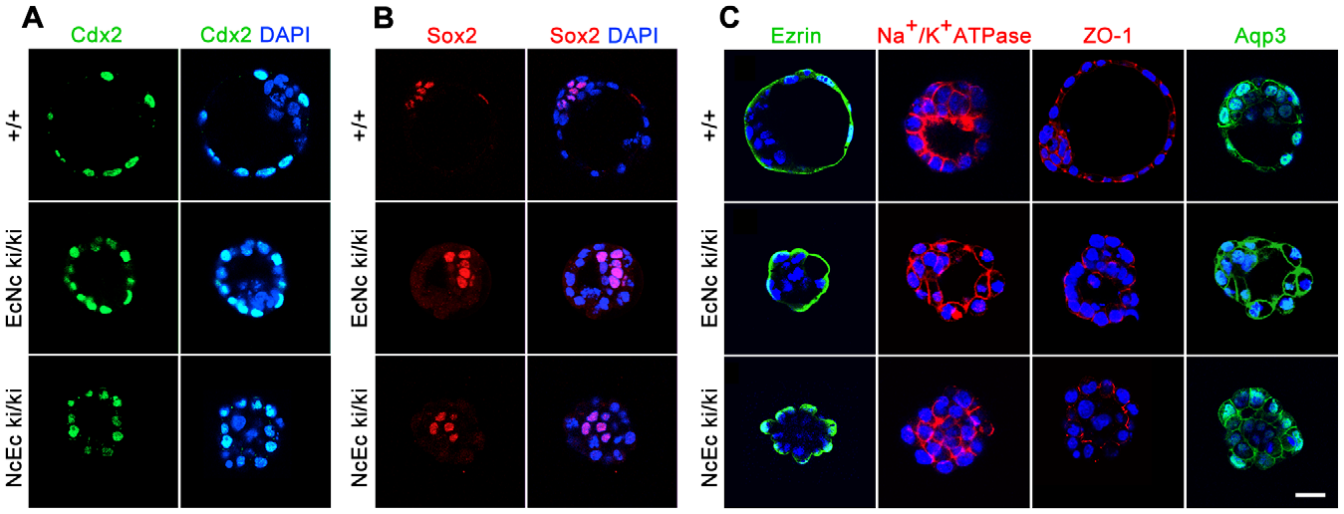
Markers for lineage specification, cell polarity, and vectorial fluid flow are correctly expressed and localized in NcEc homozygous mutants. (A) Proper segregation of outer TE cells is shown by anti-Cdx2 labeling in wt, EcNc and NcEc homozygous mutants at E3.5. (B) Wildtype, EcNc and NcEc homozygous mutant embryos labeled for the ICM cell marker Sox2 in inner cells, showing ICM cell specification and its localization inside NcEc homozygous embryos. (C) Ezrin and Na^+^/K^+^-ATPase staining to verify apical-basal polarity with same distribution in wt, EcNc and NcEc homozygous mutants at the apical and basolateral membrane. Correct sealing of the TE layer is indicated by the presence and proper localization of the tight junctional component ZO-1 at apical sites of lateral TE membranes in wt, EcNc and NcEc homozygous embryos. Key components required for vectorial fluid transport are shown by the presence of Na^+^/K^+^-ATPase and Aqp3. In embryos of all genotypes, this expression is detected in the outer cells, without any obvious differences in expression between the embryos. Hence, the first lineage segregation is specified correctly, and proteins that are essential for the TE formation process and its function are present and properly localized in NcEc homozygous mutants. Nuclei were labeled with DAPI (blue). Scale bar, 25 µm.

To confirm that lineage segregation, cell polarity and expression of molecules playing a key role during the cavitation process were not affected in NcEc homozygous embryos, we analyzed the expression of specific marker genes by immunofluorescence labeling. No difference in the expression or localization of essential proteins was found in NcEc embryos, indicating that the ICM and the TE were correctly specified and that the cavitation machinery was present (Figure 3A and 3B). Cell polarity was correctly established based on detection of apical staining of ZO-1 and Ezrin and basolateral localization of the chimeric cadherins and Na^+^/K^+^-ATPase (Figure 3C). In contrast to *E-cad^−/−^*, both EcNc ki/ki and NcEc ki/ki embryos show proper expression and membrane localization of β-catenin, Plakoglobin and p120ctn (Figure S2A). They were connected to both chimeric cadherin molecules in a similar manner as detected by immunoprecipitation (Figure S2B). In addition, embryo-derived homozygous TE cells differentiated into trophoblast giant cells, and ES cells showed proper adhesive colony formation. Similar differentiation capacities were observed for EcNc ki/ki and NcEc ki/ki genotypes, in stark contrast to *Ecad^−/−^* ES cells (Figure S3). This indicated that cell polarity, adhesion, cadherin complex composition and the cavitation machinery are well established in both homozygous mutants.

### Absence of the extracellular domain of E-cad leads to the induction of apoptosis in TE cells

**Figure 4:**
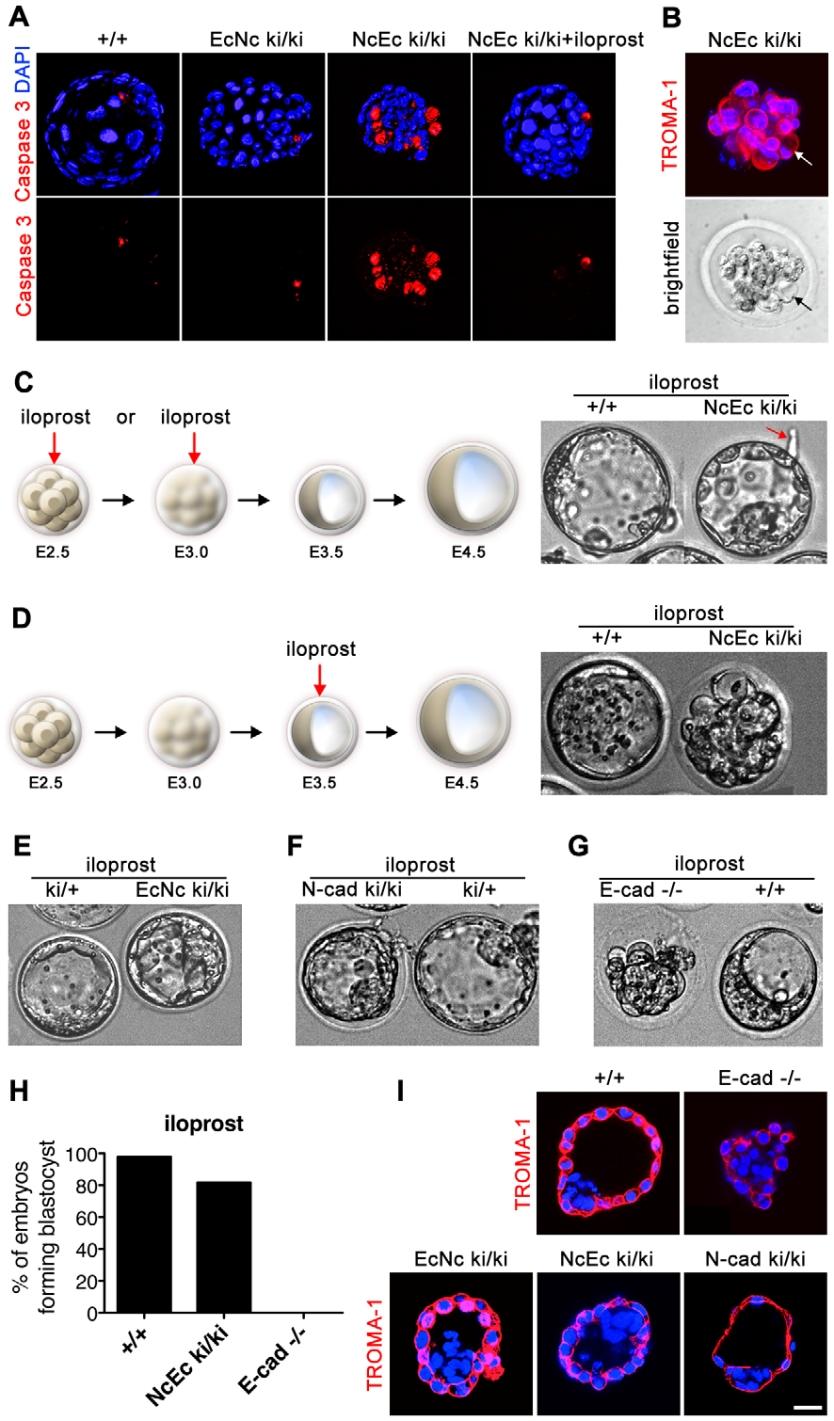
Increased apoptosis is detected in the outer cells of homozygous NcEc embryos and is blocked by iloprost treatment, rescuing blastocyst formation. (A) Labeling of cleaved and activated Caspase 3 (red) shows only one apoptotic cell in the ICM of control and EcNc ki/ki embryos, whereas the outer cells of NcEc homozygous mutant embryos display a substantial increase in Caspase 3-positive cells. Cleavage of Caspase 3 was not detected upon iloprost treatment (1 µM) (B) Cell blebbing and vacuolization is detected in TROMA-1 positive outer cells of NcEc ki/ki embryos demonstrating induction of PCD in cells destined to become TE (arrow). (C) Treatment of NcEc ki/ki embryos with 1 µM iloprost at the precompacted or compacted morula stage (E2.25–E3.0) observed by time-lapse microscopy rescues the TE formation defect and hatching is initiated (arrow). (D) Treatment with iloprost at a later time-point (E3.5) did not rescue the phenotype. (E) One representative frame of a time-lapse recording of iloprost-treated EcNc ki/ki embryos. The formation of the blastocyst is marginally improved. (F) Treatment of N-cad ki/ki embryos with iloprost resulted in a rescue similar to that for NcEc homozygous mutants. (G) *E-cad*-null embryos were not rescued upon treatment with iloprost. (H) Percentage of iloprost-treated embryos that formed a proper blastocyst in time-lapse experiments and during *in vitro* culture for wt (n = 65), NcEc homozygous mutants (n = 33) and *E-cad^−/−^* (n = 15) in >5 independent experiments. (I) With the exception of *E-cad^−/−^* embryos, proper specification of the rescued TE of iloprost-treated homozygous mutants was confirmed by cytokeratin 8 expression, which is restricted to TE cells (TROMA-1, red). Scale bar, 25 µm.

One major difference between the NcEc ki/ki embryos and the EcNc ki/ki embryos was that the failure of proper TE formation in NcEc mutants was accompanied by cell scattering and vacuolation in the outside cells, both of which indicate the induction of programmed cell death (PCD). To verify an aberrant induction of PCD in NcEc mutants, embryos were labeled for cleaved Caspase 3, a general marker for the activation of apoptosis. A substantial increase in number of Caspase 3-positive cells was detected in the TROMA-1 labeled outer cells of the mutants (Figure 4A and 4B). In contrast, no apoptosis was found in the TE cells of homozygous EcNc embryos or control littermates. Moreover, EcNc ki/ki embryos did not show a delayed onset of apoptosis as identified in prolonged embryo cultures for additional 24 h, indicating that in these mutants TE was not prone to PCD (Figure S4B and S4C). Interestingly, the induction of PCD in homozygous NcEc mutants was phenocopied if wildtype (wt) embryos were incubated with staurosporine, a bacterial-derived alcaloid which activates PCD by inducing Caspase 3. Treating wt embryos with 50 nM staurosporine severely compromised blastocyst formation (Figure S5A, S5B and S5D). This result strongly indicated that in NcEc mutants, the fragile equilibrium between cell survival and cell death was shifted towards apoptosis due to the misexpression of the NcEc chimeric cadherin in the *E-cad* (*Cdh1*) expression domain. Moreover, since increased PCD was not detected in EcNc mutants, we concluded that replacing the extracellular domain of E-cad with N-cad specifically caused this imbalance. In the following experiments, we sought to re-establish this fine-tuned equilibrium to promote cell survival.

### Blocking PCD rescues the blastocyst formation defect observed in NcEc ki/ki mutants

Proper preimplantation development in mice and humans relies on the orchestrated program of various growth factors and small secreted molecules, such as prostaglandins, which are produced by the embryo and the oviduct, [30], [31]. *In vitro*, activation of prostacyclin-dependent signaling enhances embryo survival and hatching of mouse, human and pig embryos by suppressing Caspase 3 activation via PPARδ and 14-3-3ε and by blocking cytochrome C release from the mitochondria [31]. Here, we used iloprost, a stable synthetic analogue of prostacyclin (PGI_2_) and analyzed the effect on blastocyst formation in NcEc homozygous mutants [32], [33]. Strikingly, if homozygous NcEc mutants were cultured between E2.5 and E3.5 in the presence of 1 µM iloprost, an accurate blastocoel cavity formed within the 24-h time-lapse recording (Figure 4C, 4D, 4H; Figure S5C; Video S3). Hence, blocking Caspase 3-mediated activation of PCD rescued this phenotype. We re-investigated N-cad ki/ki embryos that also failed to form a TE under standard conditions [21]. Interestingly, treatment of those embryos rescued blastocyst formation in a similar fashion, whereas there was only a moderate effect on homozygous EcNc mutants, resulting in enhanced stability of the TE (Figure 4E, 4F and Video S4). In contrast, blastocyst formation was not rescued in *in vitro* cultured *E-cad*-null embryos, presumably due to the entire absence of cadherin-mediated adhesion, which is indispensable for TE formation (Figure 4G, 4H and Video S5). The proper spatial organization and the epithelial nature of the TE in iloprost-stimulated mutants were confirmed by TROMA-1 staining. An intact TE layer of TROMA-1-positive cells that was correctly separated from TROMA-1-negative ICM cells was detected in homozygous NcEc and N-cad ki/ki mutants (Figure 4I). Manipulation of other branches of the PCD pathway resulted similarly in rescue of NcEc embryos. Either blocking p53 by cyclic pifithrin alpha (cPFT) or directly inhibiting Caspase 3 by Z-DEVD-FMK enabled both TE formation and the maintenance of blastocyst integrity (Figure S4A). These results indicated that prosurvival cues need to be active during blastocyst formation and that in homozygous NcEc mutants the shifted balance of cell survival and PCD was artificially returned to its equilibrium by inhibiting the apoptotic program at different levels. However, this rescue was only possible in a cadherin-mediated manner since blocking apoptosis rescued only cadherin-expressing embryos.

### Activation of insulin-like growth factor I receptor (Igf1r) signaling by excess Igf1 rescues blastocyst formation in NcEc embryos

**Figure 5:**
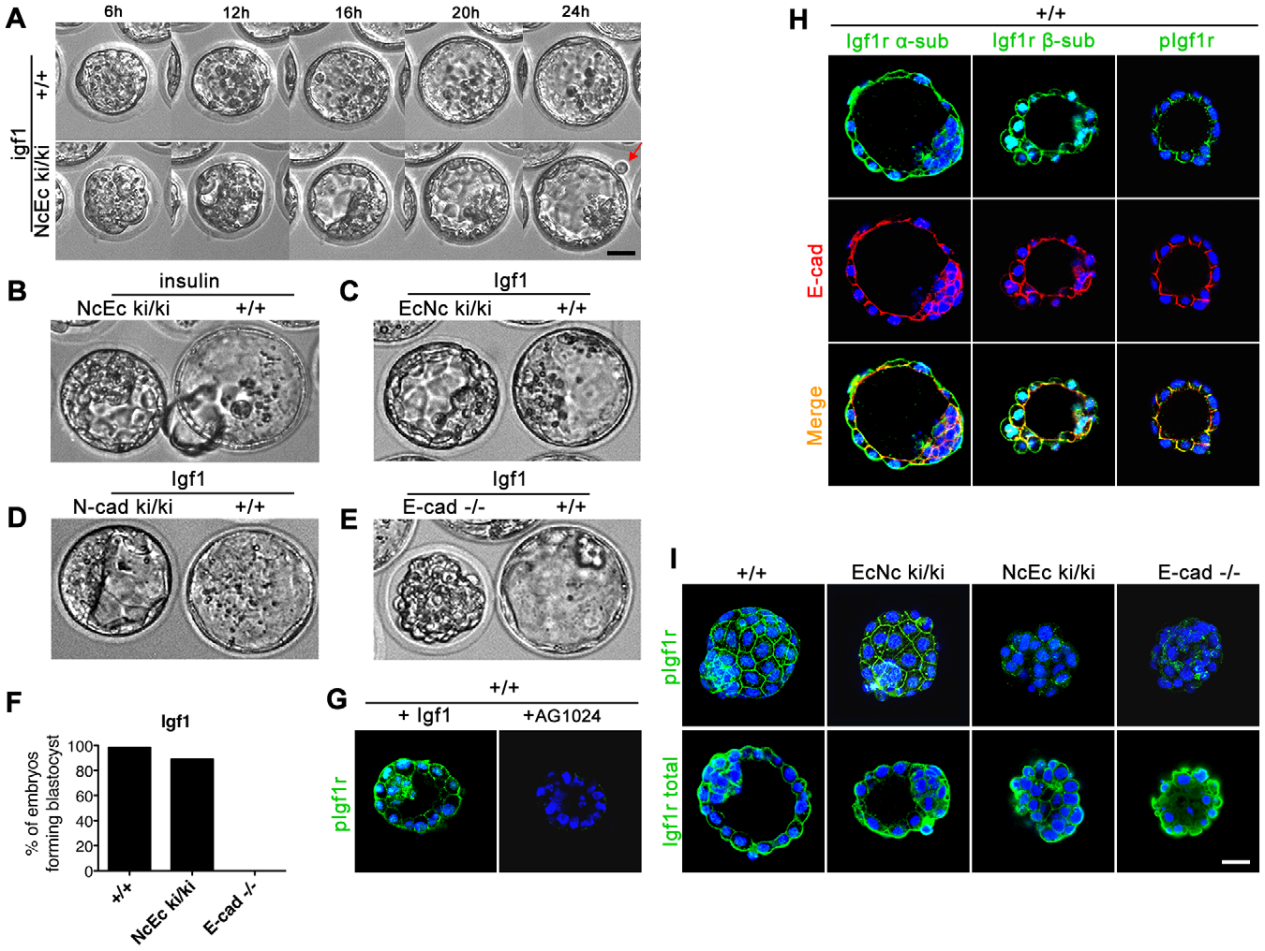
Artificial increase of Igf1 levels during *in vitro* culture rescues blastocyst formation suggesting Igf1 signaling as the endogenous prosurvival stimulus. (A) Wildtype and homozygous NcEc mutant embryos were incubated in the presence of 100 ng/ml Igf1 and recorded in 15-min intervals for 24 h. Images are displayed for 6-h intervals. Mutant embryos form a proper blastocyst similar to their control littermates and initiation of hatching is observed (arrow). (B) Insulin treatment rescues TE formation in a similar but milder fashion compared to Igf1 treatment. (C) Incubation of homozygous EcNc mutant embryos with Igf1 did not result in significant changes in blastocyst formation. (D) N-cad ki/ki embryos formed a blastocyst in the presence of Igf1. (E) *E-cad^−/−^* embryos were not rescued by Igf1 treatment. (F) Percentage of Igf1-treated embryos that formed a proper blastocyst in time-lapse experiments and during *in vitro* culture for wt (n = 31), NcEc homozygous mutants (n = 18) and *E-cad^−/−^* (n = 13) in >5 independent experiments. (G) Incubation of wt embryos with 100 ng/ml Igf1 or 10 µm Tyrphostin AG1024, a specific Igf1r inhibitor, induced hyperactivation or absence of Igf1r activation, respectively, demonstrating specificity of both the anti-Igf1r antibody and the inhibitor. Nuclear staining of the β-subunit and the phosphorylated form of Igf1r is additionally detected in the nucleus and is increasing upon Igf1 treatment as observed previously [65]. (H) The activated form of Igf1r showed protein colocalization with E-cad in the TE cells of wt embryos. An antibody detecting the α- or the β-subunit of Igf1r (total Igf1r) showed localization of Igf1r throughout the membrane, partially overlapping with E-cad (left and middle panel, respectively). A complete overlap of the activated phosphorylated form of the receptor (pIgf1r) and E-cad labeling at lateral cell-cell contact sites was detected in the TE (right panel). (I) Igf1r was hypoactivated in NcEc- and *E-cad*-null embryos. Immunofluorescence labeling of pIgf1r and total Igf1r showed comparable intensities of activated Igf1r in wt and EcNc embryos, whereas a substantial reduction was found in NcEc and *E-cad^−/−^* embryos. Total Igf1r levels were unaffected. Scale bar, 25 µm.

Since all heterozygous mutant mice analyzed here did not show defects and developed normally until adulthood, a dominant effect of an inappropriate cadherin in TE cells is very unlikely. This was confirmed by analysis of compound NcEc/EcNc mice that show proper blastocyst formation capacity (Figure S4E). When searching for a putative prosurvival signal that is triggered by the presence of the extracellular domain of E-cad, we focused on receptor tyrosine kinase (RTK) signaling cascades. In specific cellular contexts, cadherins interact with RTKs like Egfr and Fgfr2 and thus modulate downstream signaling activities [34]–[36]. Since incubation with bFGF did not improve blastocyst formation (data not shown), and mutations in *Egfr* do not reveal TE defects [37], we focused on Igf1r-mediated signaling. In previous studies, Igf1 enhanced blastocyst formation by providing a survival signal through PI3K/Akt [27], [28]. In agreement with these data, blocking Igf1r signaling in wt embryos at the morula stage with a specific inhibitor (Tyrphostin AG1024) induced cell fragmentation of outer cells and blocked TE formation (Figure S5E). When NcEc homozygous mutant embryos were treated with 100 ng/ml Igf1 for 24 h and recorded with time-lapse microscopy, these embryos formed a stable TE and even initiated hatching at the end of the recording (Figure 5A, 5F; Figure S4A, S4B; Video S6). In agreement with our previous results, Igf1-mediated rescue was observed in homozygous NcEc and N-cad ki/ki mutants but not in *E-cad^−/−^* embryos (Figure 5C–5F and Videos S8 and S9). To rule out the possibility that Igf1 simply delayed the induction of PCD we generated prolonged embryonic cultures of NcEc ki/ki. After initial 24 h incubation, embryos were transferred to fresh medium and kept for additional 24 h in the incubator. In the presence of Igf1 NcEc ki/ki embryos formed a stable TE without showing indications of apoptosis (Figure S4B). Thus, the treatment of NcEc ki/ki embryos with either Igf1, iloprost or cPFT rescued apoptosis as indicated by absence of active Caspase 3 staining (Figure S4D).

*In utero* as well as *in vitro*, preimplantation embryos receive insulin (*Ins1*)- and insulin receptor (*Insr*)-mediated signals [23]. Thus, homozygous mutants were incubated with 25 µg/ml insulin to determine whether this pathway contributes to cell survival. Although there was a significant improvement in the formation of a blastocoel cavity, the effect was modest in comparison to Igf1 treatment (Figure 5B and Video S7). This result revealed that Igf1 and the activation of its receptor Igf1r plays a crucial role during preimplantation development and the receptor kinase activity provides the endogenous survival signal in wt embryos that is blocked or attenuated in NcEc and N-cad ki/ki mutants.

### E-cadherin interacts with Igf1r and is required for efficient receptor activation to mediate the survival of TE cells

**Figure 6:**
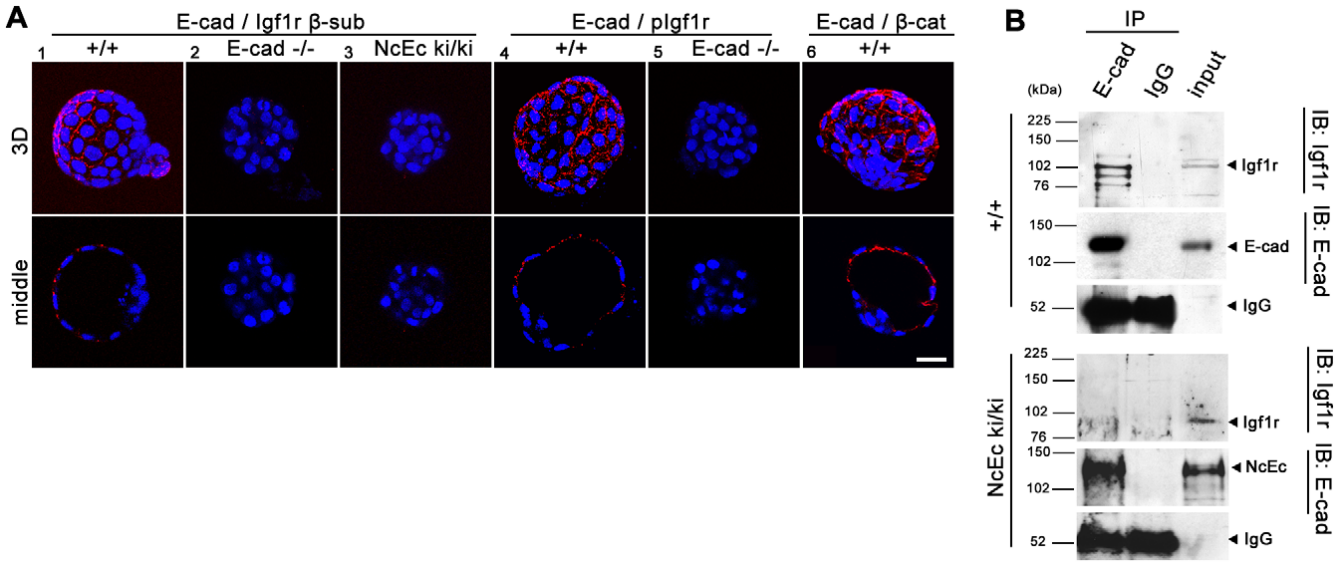
E-cad interacts with Igf1r and increases receptor activity in TE cells. (A) A proximity ligation assay (Duolink) examining wt (1, 4, 6), *E-cad*-null (2, 5) and NcEc homozygous embryos (3). Red dotted fluorescence indicates sites of interaction of analyzed proteins as indicated in optical sections (lower row) and 3D reconstructions (upper row). The E-cad-Igf1r interaction was detected by antibodies against the β-subunit of Igf1r and the intracellular domain of E-cad, which also binds to the NcEc protein. In wt embryos, fluorescent dots indicating sites of protein-protein interactions, were found at cell-cell contact sites (1), whereas *E-cad*-null embryos that contained only residual maternally derived E-cad and NcEc homozygous embryos did not show a fluorescence signal, although both anti-Igf1r and anti-E-cad antibodies detect the β-subunit of Igf1r and NcEc, respectively in NcEc homozygous mutant embryos (2, 3). Similar results were obtained in a Duolink assay using anti-E-cad (intracellular domain) and anti-pIgf1r (activated form) antibodies (4, 5). The known interaction between E-cad and β-catenin gave a punctuate pattern at basolateral membranes in cells of wt embryos as control (6). (B) The interaction between E-cad and Igf1r analyzed by co-immunoprecipitation experiments in wt and NcEc ki/ki TS cells. Immunoprecipitation with anti-E-cad and IgG control antibodies displayed a specific interaction of E-cad to coprecipitated Igf1r (total) in wt TS cells (upper panel) whereas the receptor was not co-immunoprecipitated with the NcEc protein in NcEc ki/ki TS cells (lower panel). 5% input was loaded in the last lane. Scale bar, 25 µm.

Our previous analysis indicated a functional link between E-cad and Igf1r. *In vitro*, a direct interaction between these two proteins was observed in MCF-7 cells [38], [39], but whether this interaction also influences Igf1r activity in a ligand-dependent or ligand-independent manner is unknown. To further study the putative role of Igf1r in preimplantation development and whether Igf1r kinase activity is facilitated by E-cad to regulate survival of TE cells, we first analyzed the expression of Igf1r and the amount of its activated form (pIgf1r). In wt embryos, the receptor was detected in preimplantation stages. It localized to basolateral membranes and additionally to the apical membrane of TE cells, showing a partial overlap with E-cad at cell contact sites (Figure 5H and Figure S5F). Interestingly, analysis of pIgf1r with a phospho-specific antibody revealed that the receptor was only activated at cell contact sites that showed substantial overlap with anti-E-cad staining at lateral membranes (Figure 5H and Figure S5F). Treatment of wt embryos with Igf1 hyperactivated Igf1r resulting in ectopic pIgf1r detection at apical sites, whereas blocking of receptor activation by Tyrphostin AG1024 abolished pIgf1r detection (Figure 5G and Figure S5F). Strikingly, and in contrast to wt or EcNc embryos, NcEc and *E-cad^−/−^* embryos showed weaker or absent pIgf1r staining intensities, although the overall amount of Igf1r was not changed (Figure 5I). Moreover, treatment of wt embryos with a chelating agent, like EGTA to deplete Ca^2+^-ions and to interfere with cadherin conformation and function [40] led to a decrease in pIgf1r levels mimicking the lack of Igf1r activation of homozygous NcEc mutant embryos (Figure S5G). These results suggested that the activity of the receptor is reduced in NcEc mutants due to lack of facilitation by interaction of E-cad and Igf1r. To test this idea, we performed a Duolink proximity ligation assay, a fluorescence-based method to show protein-protein interaction *in situ*. As a control, we analyzed the known interaction between E-cad and β-catenin in wt blastocysts, which showed fluorescent signals at sites of interaction at basolateral membranes as expected (Figure 6A, 6). Analysis of the interaction of E-cad and Igf1r by Duolink revealed fluorescent labeling in wt blastocysts. In agreement with the cellular distribution of pIgf1r, interaction was detected at lateral membranes (Figure 6A, 1). A similar assay in wt embryos using an antibody against pIgf1r gave a comparable result, suggesting that E-cad interacts with the activated form of the receptor or that only E-cad-bound receptor becomes activated (Figure 6A, 4). However, In NcEc ki/ki embryos only a reduced and almost absent signal was present, although anti-pIgf1r and anti-E-cad antibodies were able to detected both proteins. The analysis demonstrated a lack of interaction in NcEc ki/ki embryos in agreement with our hypothesis (Figure 6A, 3). No signal was obtained in *E-cad*-null embryos (Figure 6A, 2, 5). In a second approach complexes between endogenously expressed E-cad and Igf1r were analyzed by anti-E-cad immunoprecipitation (IP). Wt and NcEc ki/ki trophoblast stem cells (TS cells) were isolated from blastocyst outgrowths and from ES cells after transient induction of ectopic Cdx2 expression, respectively. Binding of E-cad to Igf1r was identified upon co-immunoprecipitation of lysates from wt TS cell lysates (Figure 6B, upper panel). Two additional fragments of lower molecular weight were specifically co-precipitated and detected by two individual anti-Igf1r antibodies (Figure 6B and data not shown). The additional bands were very faint in the input samples. They presumably represent γ-secretase processed forms of the receptor as observed previously [41] generated upon activation and are enriched in the immunoprecipitation (Figure 6B, upper panel). In contrast to that, no interaction was seen when chimeric NcEc was precipitated with the same anti-E-cad antibody from lysates of NcEc ki/ki TS cells. Specific Igf1r signals were not detectable in IgG and E-cad IPs. Our data suggest that E-cad and Igf1r interact in the TE at sites of cell-cell contact. This interaction is indispensable for TE formation via facilitation of RTK signaling activity, which in turn promotes cell survival and keeps apoptosis at bay.

## Discussion

**Figure 7:**
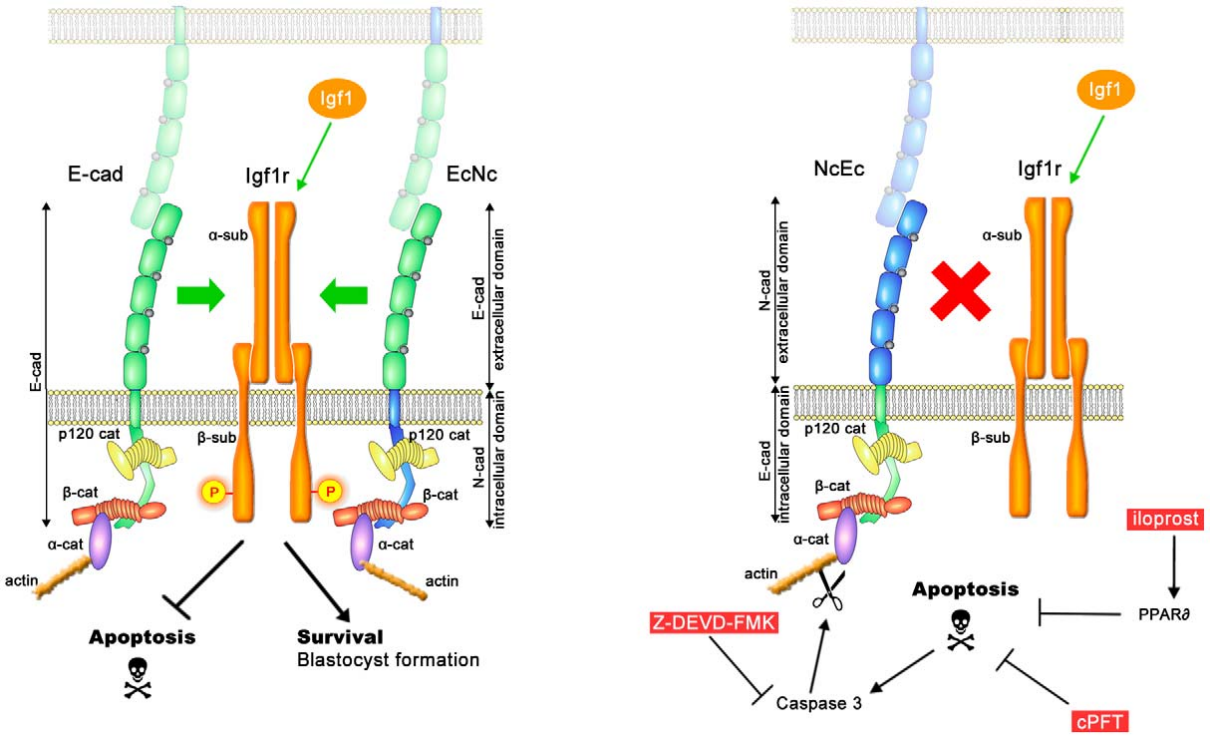
A model of the molecular pathways that are involved in TE survival but are blocked in homozygous NcEc and N-cad ki/ki mutants. In the presence of full-length E-cad or of the E-cad extracellular domain in EcNc embryos interaction of cadherins with Igf1r is occurring. This enables proper activation of Igf1r upon Igf1 signaling (phosphorylation), which supplies survival signals and blocks PCD (left panel). In the absence of E-cad cell adhesion is maintained in presence of NcEc or N-cad, but both proteins are incapable of interacting with Igf1r. As a consequence of the uncoupled interaction, Igf1r is not fully activated, prosurvival signals are lacking and apoptotic pathways reach the threshold levels for PCD induction (right panel). In the presence of cadherin-mediated adhesion (in homozygous NcEc and N-cad ki/ki, but not in *E-cad*-null embryos), apoptotic pathways can be blocked only by external cues (red boxes), which inhibit PCD at different levels and thereby rescue TE formation. According to this model E-cad is required for providing survival cues via the extracellular domain in addition to its role in cell adhesion.

Cadherins are bona fide adhesion molecules that are involved in clustering cells of the same type together. Additionally, a role in cadherin-mediated RTK signal transduction through their interactions with different receptors has been suggested. These interactions either attenuate or enhance RTK activation in a ligand-dependent or ligand-independent manner [34]–[36], [42], [43]. The Igf/insulin-like growth factor I receptor axis controls growth, differentiation and cell survival and comprises Igf1, Igf2 and insulin as ligands and Igf1r, Igf2r and insulin receptor (Insr) [23]. The activity of this pathway is further regulated by the Igf1-binding proteins Igfbp3, Igfbp4 and Igfbp5 [44], [45]. In addition, Igf1 binds to Insr and insulin to Igf1r with lower affinity, and Igf2 signaling is transduced through both Igf1r and Insr simultaneously [25]. In contrast, the major role of Igf2r is to attenuate Igf1 and Igf2 signals since it lacks a kinase domain [46]. During preimplantation development, *Igf1r*, *Igf2r* and *Insr* are expressed, and Igf1 and insulin are provided both maternally and zygotically [47]. Treatment of embryos *in vitro* with Igf1 enhances embryo viability via mitogenic and anti-apoptotic responses, indicating that Igf1 has a role in providing survival signals [27], [28], [30]. In this study, we unraveled a link between E-cad and Igf1r that promotes cell survival in the TE. Our data indicate a previously unknown function of Igf1r during preimplantation development. Furthermore, full activation of the Igf1r kinase domain likely requires physical interaction to the extracellular domain of E-cad. Abrogating cadherin function by conformational changes upon Ca^2+^-withdrawal [40], [48] results in reduced phosphorylation of Igf1r. If E-cad is replaced by either N-cad or a chimera harboring the extracellular domain of N-cad, no interaction of the two proteins is detected, and Igf1r is only inefficiently activated. Consequently, the balance between cell survival and cell death is shifted towards PCD, and embryos cannot form a functional TE. Homozygous NcEc and N-cad ki/ki embryos are rescued by an excess of Igf1 ligand because the activity of the receptor is artificially raised to normal levels. However, a full rescue of blastocyst formation is possible only if cadherin-mediated cell adhesion is also present (Figure 7). We hypothesize that E-cad, in addition to its role in mediating homophilic cell adhesion, has a novel function during preimplantation development and triggers the survival signal initiated by Igf1/Igf1r activation.

Mice in which components of the Igf axis are knocked out have been described by several groups, and deficiencies in this axis lead to reductions in body size and weight [44]. *Igf1* mutants are severely affected and display muscle dystrophies, with only 5% of offspring reaching adulthood, and these mice are infertile [26]. Although single, double and even triple knockouts of components of the Igf axis do not show preimplantation defects [25], [46], certain combinations of mutations always result in infertility. These mice may not show preimplantation defects, due to residual maternal activity: that is, the mice still receive Igf/insulin signals from maternal tissues, and they are also provided with maternal mRNA and protein for the receptors of the Igf axis during oocyte maturation. A combined maternal/zygotic loss-of function experiment has not been performed yet to show whether the complete depletion of ligands and receptors results in TE formation defects. By treatment with Tyrphostin AG1024 we here targeted Igf1r and Insr of both maternal and zygotic origin. This simultaneous blocking of entire downstream signaling results in the induction of apoptosis. In addition to previous observations, our data support the importance of Igf1r signaling already during preimplantation development, since also E-cad dependent loss of Igf1r activation results in inefficient maintenance of survival signals. Analyses of loss- and gain-of-function mutations are indicating a general underlying mechanism that controls cell survival. Cells isolated from *Igf2*-null or *Igfr1*-null animals display increased apoptosis as indicated by the small body size [25], [49]. In contrast, overexpression of *Igf1* or *Igf2* led to a decrease in apoptosis, observed as non-involuting mammary glands and pancreas hyperplasia, indicating a dramatic shift towards cell survival [50], [51]. Combined with our new findings Igf1 has a general role in controlling cell survival and cell death, a function that is active during preimplantation development as well.

Cadherin-mediated modulation of RTKs has been previously shown for Egfr and Fgfr2 [34], [35], [42], and it may be an intrinsic property of RTKs that they need to be clustered by or interact with cadherins to be efficiently activated. Interestingly, soluble E-cad isolated from the serum of cancer patients blocks apoptosis via activation of Egfr in MDCK cells [52]. This suggests a comparable role of the E-cad/Egfr interaction to that found in our analysis for Igf1r. It is tempting to speculate that during preimplantation development control of PCD by Igf1r is regulated in an E-cad-dependent manner and has an important function. The coupling of both proteins may act as a sensor to eliminate embryos that are unable to manage the crucial step during the morula to blastocyst transition, in which the embryo switches from depending on pyruvate to glucose [53]. This switch is correlated with increasing demands for ATP within the embryo, to support Na^+^/K^+^-ATPase activity, and is mediated by Igf1r [54]. In line of our hypothesis, the fragile balance between survival and apoptosis is linked to an interaction of Igf1r and E-cad, which acts as a checkpoint to assess the viability of the embryos. Saving nutrients and energy by eliminating abnormal embryos at an early stage is a favored strategy. Our analysis suggests a hitherto unknown preimplantation checkpoint that couples integrity of the TE to embryo survival. Additionally, there is evidence that connects E-cad and the regulation of the PCD-cell survival balance in other tissues. Hyperactivated Igf1r in the mammary gland shifted the balance towards cell survival [50], whereas an opposite effect that promotes apoptosis was observed after *E-cad* (*Cdh1*) depletion. During lactation milk production is hampered due to precocious involution, resulting in a shift towards PCD [55]. It will be interesting to address whether loss of E-cad also impairs proper Igf1r function in the mammary gland by a similar mechanism as described here.

Although our data are in favor of this model, we cannot fully exclude a different mechanism and contribution of secondary effects. Many RTKs utilize co-receptors like integrins or adhesion molecules, such as CD44 for proper function [56]. In our mutants adhesion and cell polarity may be altered resulting in improper localization of Igf1r. However, staining for total Igf1r and cell polarity markers of NcEc ki/ki mutants indicated that Igf1r expression and protein localization were not changed and the TE cells still maintain apical-basal polarity. Nevertheless, secondary effects may occur due to reduced cell adhesion based on the artificial nature of the chimeric cadherins and/or to altered connection to the cytoskeleton, essential for proper adhesion. This may differ between the two molecules EcNc and NcEc and may have escaped from our analysis. Interestingly, our data suggest that in addition to the extracellular domain the intercellular domain of E-cad contributes to TE formation and normal development as well since the EcNc homozygous mutants are incapable of hatching. Unique molecular features may reside in differential affinities to bind to β-catenin or other interacting proteins, as has been suggested previously [57]. E-cad and N-cad differ in their interactions with p120ctn isoforms, which may influence p120ctn-mediated small GTPase activity and the flexibility of adherens junctions [58]. Very likely, unique intracellular interaction partners exist for both cadherins but need to be identified in future experiments.

Our study has unraveled a novel role of Igf1r activity and a crucial mechanism that provides a link of how E-cad may control the balance between cell survival and PCD. Igf1r activation is essential to promote cell survival in the TE lineage, but requires E-cad-mediated facilitation of the signal via protein interaction. Unraveling the role of this function and its implication for morphogenesis and differentiation will have a significant impact on our understanding of cadherin-mediated signaling during embryogenesis and in human diseases, such as cancer.

## Materials and Methods

### Ethics statement

Animal husbandry and all experiments were performed according to the German Animal Welfare guidelines and approved by the local authorities.

### Generation of knock-in mice and genotyping

cDNAs of E-cad and N-cad were used to generate coding sequences for chimeric proteins EcNc and NcEc corresponding to the extracellular domain of E-cad (amino acids 1–710) fused to intracellular domain including transmembrane portion of N-cad (aa 725–906) and aa 1–724 of N-cad fused to aa 711–884 of E-cad, respectively. Both sequences were combined with a C-terminal HA-tag and inserted into the ATG codon of the previously described targeting vector (pBluescriptII, Stratagene) using standard molecular cloning techniques [21], [22]. Homologous recombination and analysis of surviving ES cells by Southern blot was done as described [21], [22]. Two independent clones were used for injection into blastocysts to generate chimeric mice. After backcrossing to Zp3-cre mice to delete the neomycin resistance cassette during oocyte maturation EcNc and NcEc heterozygous mouse lines were established [59], backcrossed to C57BL/6 and *inter se* to obtain homozygous mutant embryos. Genotyping was performed by PCR using tail biopsies, yolk sacs or entire embryos with the following primers: wt allele (Ecad5′UTR_s, CCC AAG AAC TTC TGC TAG AC/Ecad1_as, TAC GTC CGC GCT ACT TCA), EcNc and NcEc alleles (ENcad3′_s, AAG CTG GCG GAC ATG TAC/Ecad1_as), EcNc (Ecad_s, ATC GCC ACA CTC AAA GTG/Ncad1_as, CTG TGG CTC AGC ATG GAT), NcEc (Ncad_s, TGG AAG CTG GTA TCT ATG/Ecad2_as, TCA TCA GGA TTG GCA GGA), Ncad ki (Ecad5′UTR_s/Ncad2_as, TGG CAA GTT GTC TAG GGA).

### Embryo time-lapse microscopy and treatments

Preimplantation embryos were isolated by flushing the oviducts or uteri with M2 medium, transferred into 10 µl KSOM droplets covered with mineral oil (Fluka). Time-lapse microscopy was performed as described with minor modifications using a Zeiss Axiovert 200 M microscope equipped with Narishige manipulators, Incubator XL and Tempcontrol together with a humidifier connected to a heating stage E100 (Zeiss) at 37°C and 7.5% CO_2_ [21], [22]. Embryos were photographed every 15 min for 24 h. Zeiss AxioVision ver. 4.8 software and Uniblitz shutters were used for the acquisition of time-lapse images. Embryos were treated with specific inhibitors and growth hormones as indicated in the following concentrations: 1 µM iloprost (Cayman chemical), 30 µM cPFTalpha (Sigma), 50 µM Z-DEVD-FMK (Enzo), 1–50 nM staurosporine (Enzo), 100 ng/ml Igf1 (eBioscience), 25 µg/ml insulin (Sigma), 10 µM Tyrphostin AG1024 (Alexis biochemicals), 2 mM EGTA. Each experiment was repeated at least five times.

### Immunofluorescence labeling and confocal microscopy

After isolation embryos were washed in PBT (0.05% Tween/PBS) and fixed with 2% PFA/PBS for 10 min. Cellular permeabilization was carried out for 5 min with 0.3% Triton X-100/PBS and embryos were incubated in primary antibody in 2.5% BSA/PBT for 2 h to overnight at room temperature. Subsequently, alexa488 or alexa594-conjugated secondary antibodies were applied for 1 h. Embryos were stained with DAPI to visualize nuclei (1∶1000, Invitrogen) and mounted in PBS droplets covered with mineral oil in glass bottom petri dishes (Willco wells). Confocal microscopy was performed using Leica TCS SP2 laser scan head attached to a Leica DM IRE2 inverted microscope. Images were processed using IMARIS software (Bitplane). Antibodies: anti-E-cadherin (intracellular), anti-N-cadherin, anti-β-catenin, anti-Plakoglobin, (BD Bioscience), HA.11 (Covance), anti-Ezrin, anti-cleaved Caspase 3 (Cell Signaling), anti-E-cadherin (extracellular, gp84) [60], TROMA-1 [61], anti-p120ctn, anti-ZO-1 (Zymed), anti-Na^+^/K^+^-ATPase (Millipore), anti-AQP3, anti-Sox2 (Calbiochem), anti-Oct4 (Santa Cruz), anti-Nanog [62], anti-Cdx2 (Biogenex), anti-Igf1r (α-subunit, abcam), anti-Igf1r (β-subunit, Cell Signaling), anti-pIgf1r (abcam).

### Duolink assay

The Duolink assay (Olink Bioscience) was performed according to the manufacturers instructions in 10 µl droplets covered with mineral oil at 37°C.

### Immunoblotting and immunoprecipitation

Immunoblotting and immunoprecipitation (IP) was performed as described for ES cell lysates (500 ng protein, 500 ng antibody) or with minor modifications for TS cells [22]. Briefly, TS cells were stimulated with 50 ng/ml Igf1 for 10 min, incubated in crosslinking buffer (6 mM KCl, 2 mM Bissulfosuccinimidyl suberate/PBS) for 30 min at 4°C, followed by quenching in 100 mM Glycine/PBS and harvested in lysis buffer (20 mM Tris-HCl pH 7.9, 137 mM NaCl, 2 mM MgCl_2_, 5 mM EDTA, 1 mM EGTA, 1% Triton X-100, 10% Glycerol, 10 mM Na_3_VO_4_, 10 mM NaF, 1× Complete protease inhibitor, Roche, 1 mM PMSF) [43]. For IP, 2 mg of protein were incubated overnight at 4°C using 1 µg anti-E-cad (BD) antibody and 25 µl slurry of protein-G coupled Dynabeads (Invitrogen). After washing in washing buffer (25 mM Tris-HCl pH 7.4, 100 mM NaCl, 0.1% Tween-20) IP samples were separated by 8–10% SDS-PAGE. Proteins of the IP or from whole cell lysates were transferred to nitrocellulose membranes using a semi-dry electroblotter (Biorad) with 200 mA for 45 min. After blocking (2% dry milk in TBS/0.1% Tween-20) for 30 min, membranes were incubated with antibody solution for 2 h to overnight and subsequently with a secondary horseradish peroxidase-conjugated antibody for 1 h. Anti-Igf1r (β-subunit), anti-Igf1r (total, α-IR3, Calbiochem), anti-E-cad (BD) and anti-Gapdh (Calbiochem) were used. Proteins were detected by soaking membranes in ECL Plus (GE Healthcare) exposed to autoradiography films.

### ES/TS-cell derivation and teratoma formation

Generation of homozygous EcNc and NcEc ES cells was performed as described previously using morulae or blastocysts from heterozygous intercrosses, plated on mytomycin-treated embryonic fibroblasts [22]. Alkaline phosphatase (AP) staining of isolated ES cells was used to verify undifferentiated pluripotent status. Cells were fixed in 4% PFA/PBS for 15 min, washed two times with PBS and incubated for 30 min in AP staining solution (25 mM Tris-maleic acid pH 9.0, 0.4 mg/ml α-naphtyl phosphate, 1 mg/ml Fast Red TR Salt, 8 mM MgCl_2_, 0.01% Na-deoxycholate, 0.02% NP40). Wt TS cells were generated similar to ES cells by blastocyst outgrowth in RPMI 1640, 20% FCS, 2 mM glutamine, 1 mM pyruvate, 50 µg/ml penicillin/streptomycin, 100 µM β-mercaptoethanol, 25 ng/ml FGF4 (Sigma), 1 µg/ml Heparin as described [63]. NcEc ki/ki TS cells were derived from transdifferentiated ES cells by stable transfection of an inducible Cdx2 plasmid (Cdx2ER) and treated with 1 µg/ml 4-OH-tamoxifen for 10 days [64]. Teratoma formation was induced by subcutaneous injection of 1×10^7^ trypsinized ES cells into BALB/c nude mice as described [21], [22].

### Paraffin embedding and immunohistochemistry

Specimen were fixed at 4°C overnight in 4% PFA/PBS and dehydrated in 30%, 50%, 70%, 100% Ethanol/PBS series for 1 h each, followed by two 10 min incubations in 100% Xylene before transferring them to paraffin overnight. Casted into paraffin blocks samples were sectioned using a RM2155 microtome (Leica) at 7 µm and stored at 4°C until further processing. Hematoxylin/eosin (H&E) staining and immunohistochemistry was carried out as described previously using epitope retrieval by 20 min boiling in Tris-EDTA pH 9.0 buffer [22], [57].

### RNA isolation and quantitative RT–PCR

Analysis of transcripts of the knock-in alleles was carried out as described previously [22]. For detection of individual transgenic transcripts the following primers were used to detect sequences of: 5′ E-cad wt or knock-in (5′EcadUPL_s, AGT GTT TGC TCG GCG TCT/5′EcadUPL_as, GCA AAG CCA TGA GGA GAC C); 3′ E-cad knock-in (3′EcadUPL_s, CAC CCC CTT ACG ACT CTC TG/HA-UPL_as, GAC GTC ATA AGG ATA TCC AGC A); 5′ N-cad knock-in (5′NcadKIUPL_s, CCA TGG CCA CTA GTA TGT GC/5′NcadKIUPL_as, AAT TTC ACC AGA AGC CTC CA); 3′ N-cad knock-in (3′NcadKIUPL_s, GGC CTT AAA GCT GCT GAC AA/3′NcadKIUPL_as, AAC CAT TAT AAG CTG CAA TAA ACA A); *Actb* (bActUPL_s, AAG GCC AAC CGT GAA AAG AT/bActUPL_as, GTG GTA CGA CCA GAG GCA TAC).

## Supporting Information

Figure S1 Detailed expression analysis of the EcNc and NcEc alleles. (A) Quantitative expression analysis of EcNc and NcEc alleles in homozygous ES cells (EcNc/EcNc and NcEc/NcEc, respectively), compared with wt (+/+), N-cad ki/ki (Nc/Nc) and Ecad-HA ki/ki (EcHA/EcHA) ES cells [21], [22]. Transcripts were compared using primers specific for the knock-in alleles [5′ E-cad (including wt allele, dark green), 3′ E-cad (light green), 5′ N-cad (dark blue) and 3′ N-cad (light blue)]. Transcript amounts in wt ES cells were set to 100% (5′ Ec). To directly compare values between different ES-cell lines and primers, values of either EcHA/EcHA or Nc/Nc of additional primer sets were set to 33%, based on the results from 5′ E-cad PCR (n.d., not detected). As observed previously, expression of the control E-cad-HA knock-in allele was reduced to 30% of wt levels. The corresponding embryos formed blastocysts, implanted and gastrulated normally [22]. Similarly, all other homozygous knock-in ES cells showed 30–45% of wt expression level. Since all homozygous ES cells showed comparable expression levels, the homozygous mutant embryonic phenotypes are attributed to differences in cadherin quality rather than in quantity. (B–D) Immunohistochemical staining of wt (B), heterozygous EcNc (C) and NcEc embryos (D) at E14.5 using anti-HA and anti-E-cad labeling, as indicated. Wildtype embryos did not show background anti-HA labeling but display E-cad expression in known E-cad expression domains. Both heterozygous EcNc and NcEc embryos showed proper expression of the HA-tagged protein in a perfect overlap with anti-E-cad staining. For higher magnification, lung and gut sections are shown as an example. The generated knock-in alleles accurately recapitulate endogenous E-cad expression. Scale bars: 1 mm (overview), 200 µm (close-up).
(TIF)

Figure S2 Expression and localization of cadherin-associated proteins are normal in homozygous NcEc embryos. (A) Wildtype (first panel), EcNc ki/ki (second panel), NcEc ki/ki (third panel) and *E-cad^−/−^* embryos (last panel) were immunofluorescently labeled with anti-β-catenin (top row), anti-p120ctn (middle row) and anti-Plakoglobin (bottom row) antibodies. Staining intensities were similar for wt and homozygous knock-in embryos and proteins properly localized to the basolateral membranes, whereas a substantial reduction of membrane localization was found in *E-cad^−/−^* embryos. This indicates that cell polarity was properly established and cadherin-associated proteins are normally distributed in homozygous NcEc mutants. (B) Immunoprecipitation experiments confirm proper adhesion complex formation in EcNc ki/ki and NcEc ki/ki ES cells. Cell lysates of the indicated genotypes were used for immunoprecipitation (IP) with IgG control, α-catenin and β-catenin (upper panel) or with anti-HA antibodies (lower panel) and immunoblotted together with 5% input to detect co-precipitated proteins as indicated. The levels of co-precipitated proteins for EcNc ki/ki and NcEc ki/ki cells are comparable, further supporting proper function of the chimeric proteins as cadherin adhesion molecules. Detected proteins are indicated by an arrowhead, asterisks label detection of IgG heavy chain and molecular weights of a molecular weight standard are given in kDa. Note, that incomplete lysis of the *E-cad^−/−^* sample results in high background in the anti-HA immunoblot and unspecific binding of β-catenin to a control IgG. Scale bar, 25 µm.
(TIF)

Figure S3 Analysis of lineage segregation and differentiation potential of ES cells derived from homozygous mutant blastocysts. Correct lineage specification of homozygous NcEc mutant embryos was further confirmed by blastocyst outgrowth and differentiation experiments of established ES-cell lines. (A) Homozygous NcEc embryos attached to a feeder cell layer and formed proper blastocyst outgrowths showing TE cells differentiating into trophoblast giant cells as indicated by cytokeratin 8 (TROMA-1) labeling. (B) From these outgrowths ES cells were established forming compact colonies in contrast to *E-cad^−/−^* ES cells that grow separated with weak or absent cell-cell adhesion. (C, D) ES cells show alkaline phosphatase activity (C) and expression of pluripotency markers Oct4, Nanog and Sox2 (D) that are similar for all genotypes. (E) 1×10^7^ ES cells injected into immunocompromised mice induced teratoma formation. In H&E stained sections of such tumors proper ES-cell differentiation capacity is confirmed by the presence of cell types derived from all three germ-layers. Homozygous mutant NcEc ES cells have comparable capacity as the corresponding control cells and formed e. g. neuroepithelium, columnar epithelium, cartilage and muscle. (F) Epithelial identity was confirmed by co-labeling of consecutive sections with anti-HA and TROMA-1 antibodies to verify expression of the chimeric cadherins in the E-cad expression domain and simultaneously expression of cytokeratin 8 (TROMA-1 positive). Scale bars, 50 µm in (A), 200 µm in (B, C, E, F) and 25 µm in (D).
(TIF)

Figure S4 Summary of different treatments to block apoptosis in NcEc and N-cad ki/ki embryos. (A) Except for *E-cad*-null embryos blastocyst formation is accomplished in the presence of 1 µM iloprost as shown in Figure 4. 30 µM of the specific p53 inhibitor cyclic pifithrin alpha (cPFT) inhibited PCD. A similar effect as with iloprost treatment was observed with embryos of the various genotypes. A specific inhibitor of Caspase 3 activation is the pharmacological compound Z-DEVD-FMK. Incubating embryos in 50 µM Z-DEVD-FMK rescued blastocyst formation, although the rescue was moderate. Summary of Igf1 treatment (100 ng/ml) of the various genotypes as shown in Figure 5 is given at the bottom. Efficiency of the rescue was observed best with Igf1 and decreased gradually in treatments with iloprost, cPFT and Z-DEVD-FMK which is correlated with the nature of the compounds acting at more downstream positions of the PCD pathway. (B) TE integrity is maintained in expanded blastocysts derived from prolonged culture of homozygous EcNc ki/ki embryos and NcEc ki/ki embryos treated with Igf1. Embryos were isolated at E2.5 and observed for 24 h for blastocyst formation. After removal of *zona pellucida* and transfer to fresh medium embryos were cultured for additional 24 h to detect expansion. Embryos formed a properly expanded blastocyst with the exception of untreated NcEc ki/ki and *E-cad^−/−^* embryos that did not show signs of cavity formation. (C) Prolonged *in vitro* culture of EcNc ki/ki embryos does not induce PCD. Untreated EcNc and NcEc homozygous embryos were stained for active Caspase 3 after prolonged culture to monitor apoptosis in 3D reconstructed confocal images. Whereas many active Caspase 3-positive cells were detected in NcEc ki/ki embryos, no increase in apoptotic cells are present in EcNc ki/ki. (D) Upon treatment of homozygous mutants by either iloprost, cPFT or Igf1 between E2.5 and E3.5, blastocyst formation was accomplished and active Caspase 3 immunoreactivity (red) decreased in homozygous mutant embryos to levels of untreated wt blastocysts. (E) Compound mice carrying one EcNc and one NcEc allele form proper blastocysts and only one or two nuclei labeled by anti-active Caspase 3, confirming that the failure of TE formation in homozygous NcEc mutants was not caused by a dominant-negative effect of NcEc. Scale bar, 25 µm.
(TIF)

Figure S5 Staurosoporine and Tyrphostin AG1024 treatment and additional Igf1r expression analysis in wt embryos. (A) Staurosporine is a chemical compound that is inducing cleavage and activation of Caspase 3 and in turn leads to PCD. Wildtype embryos that have been incubated with 10 nM or 50 nM staurosporine were incapable of forming a proper blastocyst due to activation of Caspase 3. They underwent PCD within 24 h (middle and right), whereas 1 nM staurosporine had no effect (left). (B) 50 nM staurosporine treatment of wt embryos activated Caspase 3 as indicated by immunofluorescence labeling with an antibody recognizing the cleaved form of Caspase 3. (C) Pre-treatment of wt embryos with 1 µM iloprost for 24 h prevented staurosporine-induced apoptosis since iloprost is antagonizing the mode of activation of staurosporine. Double-treated embryos form a blastocyst similar to DMSO-treated controls. (D) Wildtype embryos treated by staurosporine show activation of Caspase 3 and phenocopied NcEc homozygous mutants. Upon Caspase 3 activation fragmented actin was detected by anti-Fractin labeling (red) and E-cad (green) was no longer detected in outside cells (arrows), whereas the ICM was more protected from the treatment. (E) Blocking Igf1r signaling prevents blastocyst formation in wt embryos. Embryos at E3.0 (top row) were treated with a specific inhibitor of Igf1r (Tyrphostin AG1024) and incubated for 24 h. Control embryos (left panel) form blastocoel cavities whereas treated littermates were incapable in forming a blastocyst, outside cells became fragmented and showed clear signs of apoptosis (right panel). (F) Distribution of total and active Igf1r shows partial overlap with E-cad. 3D reconstruction of z-stacks of same embryos as shown in Figure 5F and 5G. Labeling with antibodies detecting the α- and the β-subunit of Igf1r show localization of Igf1r throughout the membrane, partially overlapping with E-cad (first and second row, respectively). Overlap of the phosphorylated activated form of Igf1r (pIgf1r) and E-cad was found at lateral cell-cell contact sites of TE cells (third row). 100 ng/ml Igf1 treatment of wt embryos induced hyperactivation detected by enhanced fluorescent signal of anti-pIgf1r labeling to ectopic apical sites (fourth row) whereas 10 µm Tyrphostin AG1024 treatment led to absence of Igf1r activation (last row). (G) Abrogating E-cad function results in strong reduction of activated Igf1r levels. Ca^2+^ was depleted by incubation of wt blastocysts with 2 mM EGTA for 15–30 min to modify tertiary structure of E-cad. Embryos were stained for pIgf1r and analyzed by confocal microscopy. Scale bar, 25 µm.
(TIF)

Video S1 Twenty-four-hour time-lapse movie with pictures taken at 15-min intervals of E2.5 precompacted homozygous EcNc morula together with control littermate (Figure 2A). Blastocyst formation in the mutant was accomplished.
(MOV)

Video S2 Twenty-four-hour time-lapse movie with pictures taken at 15-min intervals of E2.5 precompacted homozygous NcEc morula together with control littermate (Figure 2B). Blastocyst formation in the mutant was hampered and outside cells lost cell-cell contacts.
(MOV)

Video S3 Twenty-four-hour time-lapse movie with pictures taken at 15-min intervals of E2.5 homozygous NcEc mutant and control littermate treated with 1 µM iloprost (Figure 4A). Blastocyst formation was rescued and hatching initiated at the end of the movie.
(MOV)

Video S4 Twenty-four-hour time-lapse movie with pictures taken at 15-min intervals of E2.5 N-cad ki/ki mutant and control embryos treated with 1 µM iloprost (Figure 4D). Blastocyst formation was rescued.
(MOV)

Video S5 Twenty-four-hour time-lapse movie with pictures taken at 15-min intervals of E2.5 *E-cad*-null and control littermate treated with 1 µM iloprost (Figure 4E). No rescue in blastocyst formation was observed.
(MOV)

Video S6 Twenty-four-hour time-lapse movie with pictures taken at 15-min intervals of E2.5 homozygous NcEc mutant and control littermate treated with 100 ng/ml Igf1 (Figure 5A). Efficient rescue of the mutants was detected with Igf1 treatment resulting in a stable TE layer resulting in hatching at the end of the movie.
(MOV)

Video S7 Twenty-four-hour time-lapse movie with pictures taken at 15-min intervals of E2.5 homozygous NcEc mutant and control littermate treated with 25 µg/ml insulin (Figure 5B). A moderate rescue with a less stable TE layer was detected.
(MOV)

Video S8 Twenty-four-hour time-lapse movie with pictures taken at 15-min intervals of E2.5 N-cad ki/ki mutant and control littermate treated with 100 ng/ml Igf1 (Figure 5D). Treatment rescued also N-cad ki/ki embryos.
(MOV)

Video S9 Twenty-four-hour time-lapse movie with pictures taken at 15-min intervals of E2.5 *E-cad*-null and control littermate treated with 100 ng/ml Igf1 (Figure 5E). *E-cad*-deficient embryos were not rescued with any treatment procedure.
(MOV)
