# Supplementary figures and images for: Igf1r Signaling Is Indispensable for Preimplantation Development and Is Activated via a Novel Function of E-cadherin
Source: PLoS Genet. 2012 Mar 29;8(3):e1002609. doi: 10.1371/journal.pgen.1002609 (PMC3315466; doi:10.1371/journal.pgen.1002609)

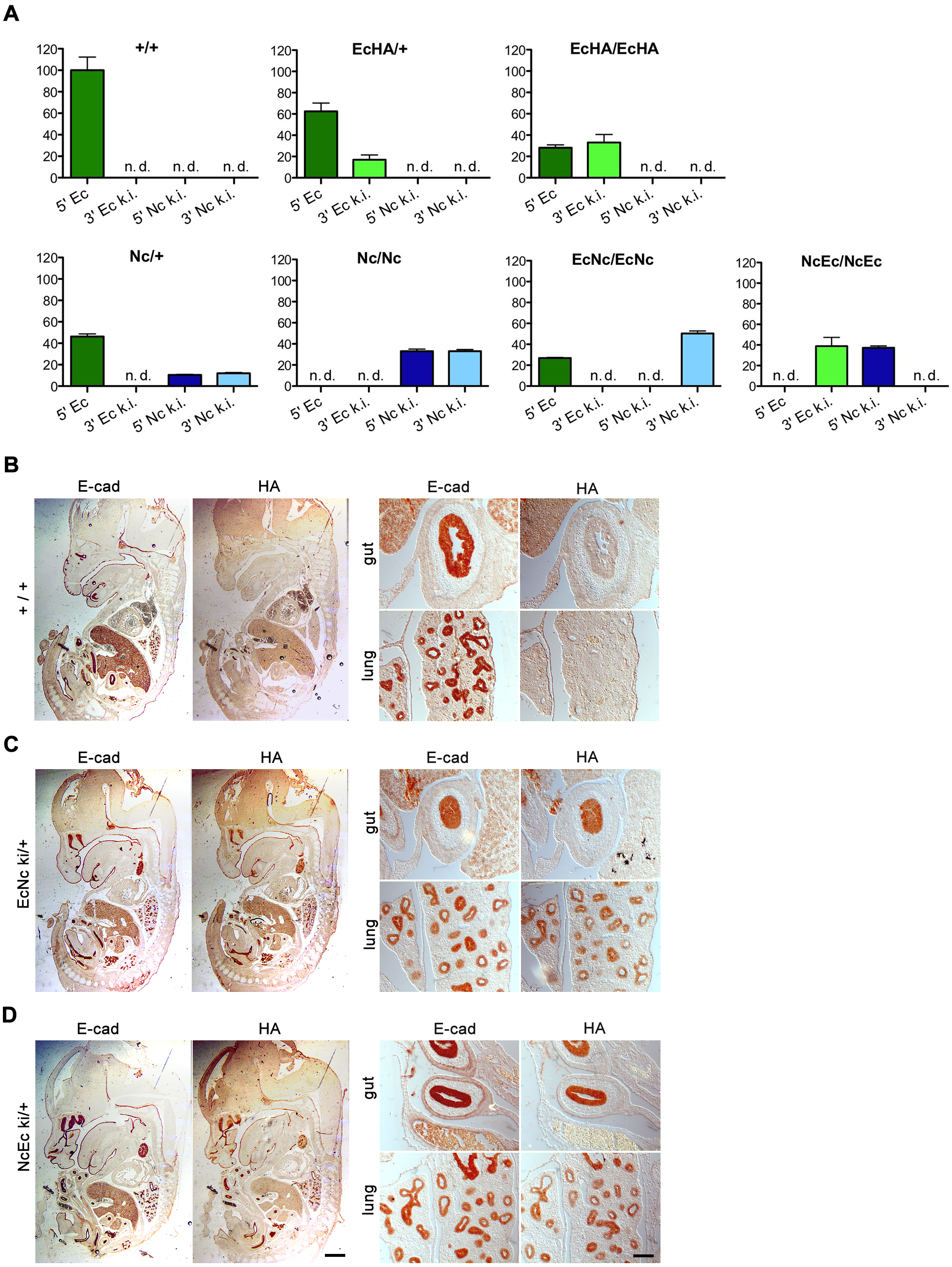

Supplement: Figure S1 — Detailed expression analysis of the EcNc and NcEc alleles. (A) Quantitative expression analysis of EcNc and NcEc alleles in homozygous ES cells (EcNc/EcNc and NcEc/NcEc, respectively), compared with wt (+/+), N-cad ki/ki (Nc/Nc) and Ecad-HA ki/ki (EcHA/EcHA) ES cells [21], [22]. Transcripts were compared using primers specific for the knock-in alleles [5′ E-cad (including wt allele, dark green), 3′ E-cad (light green), 5′ N-cad (dark blue) and 3′ N-cad (light blue)]. Transcript amounts in wt ES cells were set to 100% (5′ Ec). To directly compare values between different ES-cell lines and primers, values of either EcHA/EcHA or Nc/Nc of additional primer sets were set to 33%, based on the results from 5′ E-cad PCR (n.d., not detected). As observed previously, expression of the control E-cad-HA knock-in allele was reduced to 30% of wt levels. The corresponding embryos formed blastocysts, implanted and gastrulated normally [22]. Similarly, all other homozygous knock-in ES cells showed 30–45% of wt expression level. Since all homozygous ES cells showed comparable expression levels, the homozygous mutant embryonic phenotypes are attributed to differences in cadherin quality rather than in quantity. (B–D) Immunohistochemical staining of wt (B), heterozygous EcNc (C) and NcEc embryos (D) at E14.5 using anti-HA and anti-E-cad labeling, as indicated. Wildtype embryos did not show background anti-HA labeling but display E-cad expression in known E-cad expression domains. Both heterozygous EcNc and NcEc embryos showed proper expression of the HA-tagged protein in a perfect overlap with anti-E-cad staining. For higher magnification, lung and gut sections are shown as an example. The generated knock-in alleles accurately recapitulate endogenous E-cad expression. Scale bars: 1 mm (overview), 200 µm (close-up). (TIF) [file pgen.1002609.s001.tif]

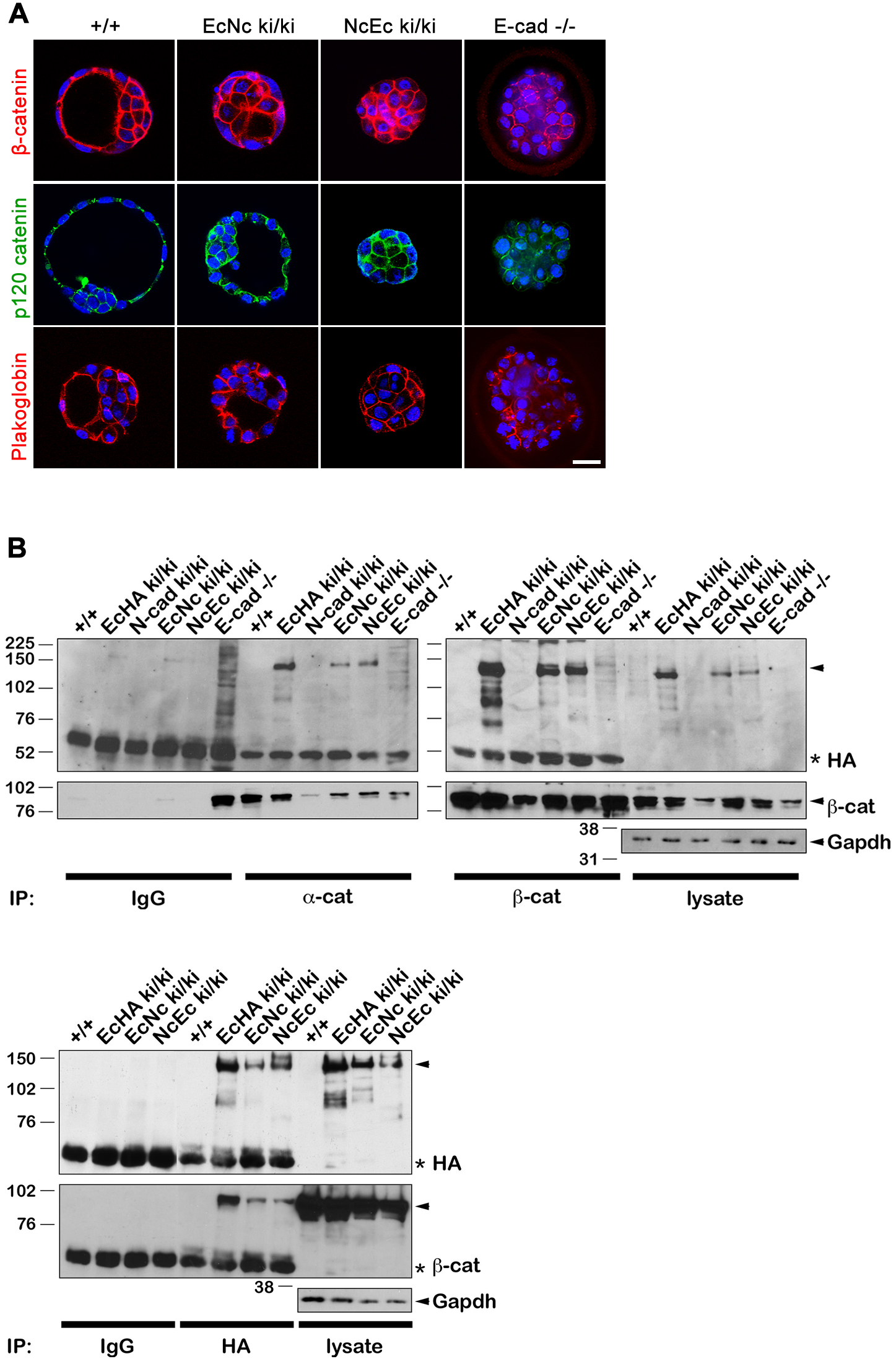

Supplement: Figure S2 — Expression and localization of cadherin-associated proteins are normal in homozygous NcEc embryos. (A) Wildtype (first panel), EcNc ki/ki (second panel), NcEc ki/ki (third panel) and E-cad−/− embryos (last panel) were immunofluorescently labeled with anti-β-catenin (top row), anti-p120ctn (middle row) and anti-Plakoglobin (bottom row) antibodies. Staining intensities were similar for wt and homozygous knock-in embryos and proteins properly localized to the basolateral membranes, whereas a substantial reduction of membrane localization was found in E-cad−/− embryos. This indicates that cell polarity was properly established and cadherin-associated proteins are normally distributed in homozygous NcEc mutants. (B) Immunoprecipitation experiments confirm proper adhesion complex formation in EcNc ki/ki and NcEc ki/ki ES cells. Cell lysates of the indicated genotypes were used for immunoprecipitation (IP) with IgG control, α-catenin and β-catenin (upper panel) or with anti-HA antibodies (lower panel) and immunoblotted together with 5% input to detect co-precipitated proteins as indicated. The levels of co-precipitated proteins for EcNc ki/ki and NcEc ki/ki cells are comparable, further supporting proper function of the chimeric proteins as cadherin adhesion molecules. Detected proteins are indicated by an arrowhead, asterisks label detection of IgG heavy chain and molecular weights of a molecular weight standard are given in kDa. Note, that incomplete lysis of the E-cad−/− sample results in high background in the anti-HA immunoblot and unspecific binding of β-catenin to a control IgG. Scale bar, 25 µm. (TIF) [file pgen.1002609.s002.tif]

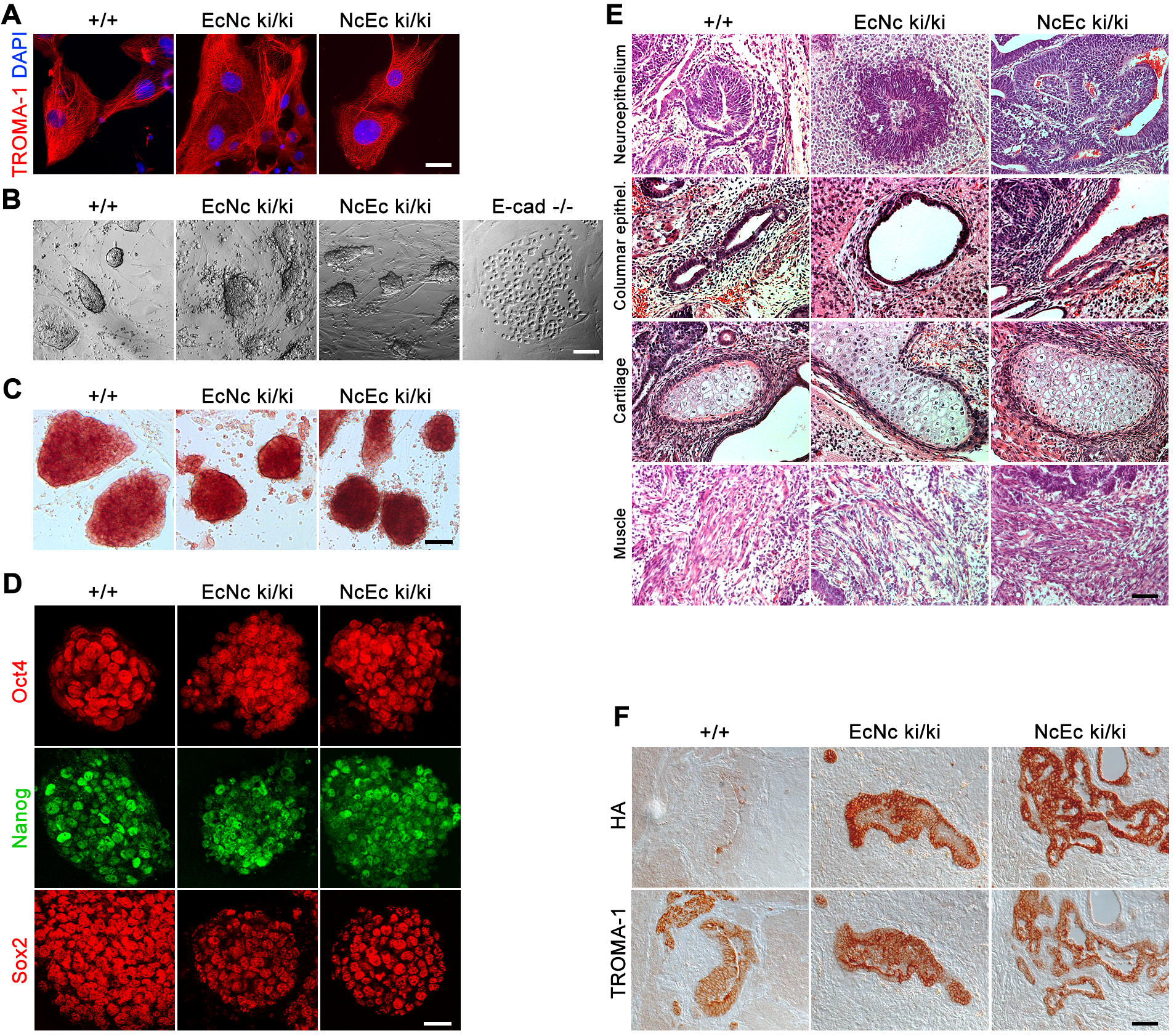

Supplement: Figure S3 — Analysis of lineage segregation and differentiation potential of ES cells derived from homozygous mutant blastocysts. Correct lineage specification of homozygous NcEc mutant embryos was further confirmed by blastocyst outgrowth and differentiation experiments of established ES-cell lines. (A) Homozygous NcEc embryos attached to a feeder cell layer and formed proper blastocyst outgrowths showing TE cells differentiating into trophoblast giant cells as indicated by cytokeratin 8 (TROMA-1) labeling. (B) From these outgrowths ES cells were established forming compact colonies in contrast to E-cad−/− ES cells that grow separated with weak or absent cell-cell adhesion. (C, D) ES cells show alkaline phosphatase activity (C) and expression of pluripotency markers Oct4, Nanog and Sox2 (D) that are similar for all genotypes. (E) 1×10^7^ ES cells injected into immunocompromised mice induced teratoma formation. In H&E stained sections of such tumors proper ES-cell differentiation capacity is confirmed by the presence of cell types derived from all three germ-layers. Homozygous mutant NcEc ES cells have comparable capacity as the corresponding control cells and formed e. g. neuroepithelium, columnar epithelium, cartilage and muscle. (F) Epithelial identity was confirmed by co-labeling of consecutive sections with anti-HA and TROMA-1 antibodies to verify expression of the chimeric cadherins in the E-cad expression domain and simultaneously expression of cytokeratin 8 (TROMA-1 positive). Scale bars, 50 µm in (A), 200 µm in (B, C, E, F) and 25 µm in (D). (TIF) [file pgen.1002609.s003.tif]

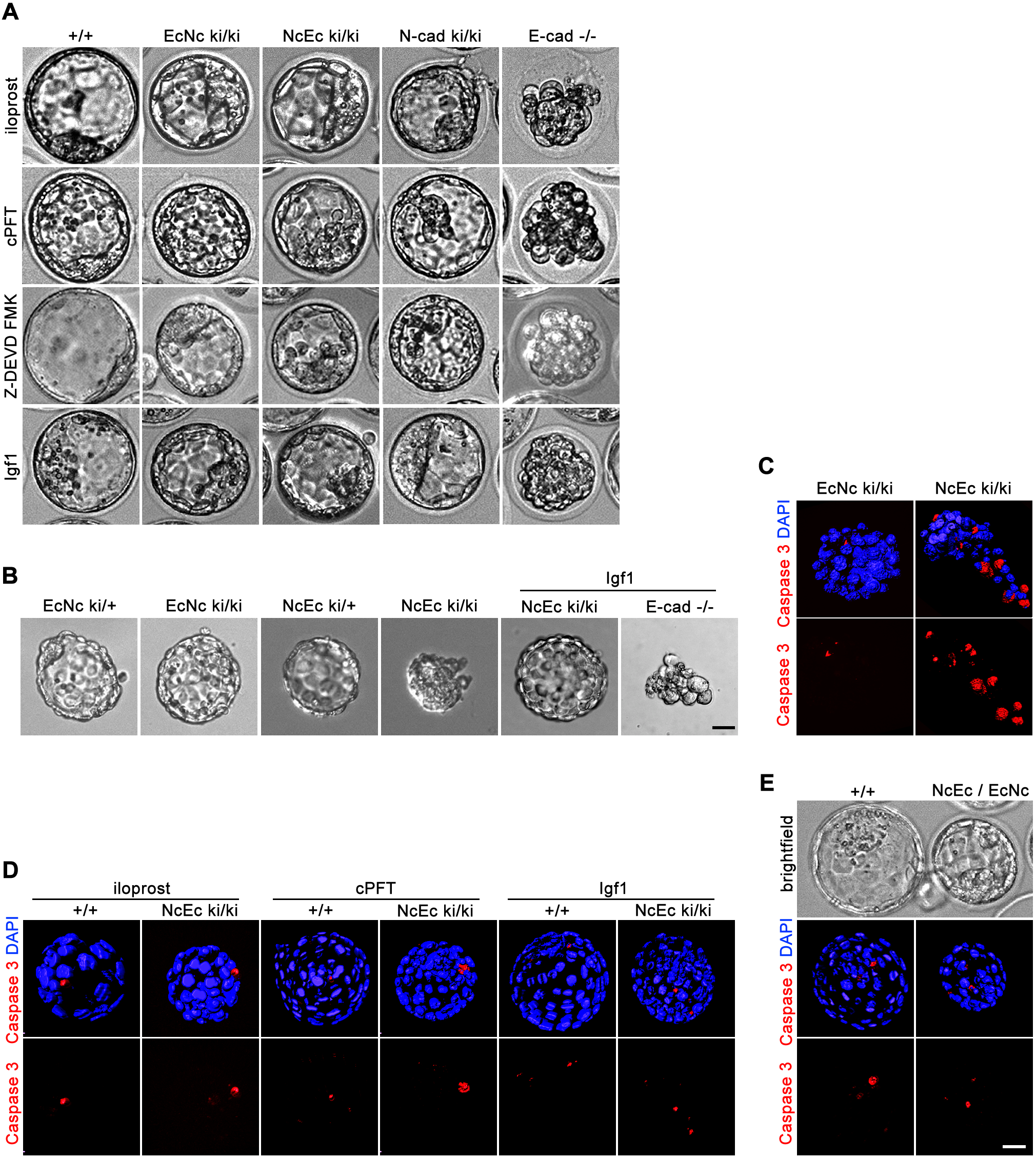

Supplement: Figure S4 — Summary of different treatments to block apoptosis in NcEc and N-cad ki/ki embryos. (A) Except for E-cad-null embryos blastocyst formation is accomplished in the presence of 1 µM iloprost as shown in Figure 4. 30 µM of the specific p53 inhibitor cyclic pifithrin alpha (cPFT) inhibited PCD. A similar effect as with iloprost treatment was observed with embryos of the various genotypes. A specific inhibitor of Caspase 3 activation is the pharmacological compound Z-DEVD-FMK. Incubating embryos in 50 µM Z-DEVD-FMK rescued blastocyst formation, although the rescue was moderate. Summary of Igf1 treatment (100 ng/ml) of the various genotypes as shown in Figure 5 is given at the bottom. Efficiency of the rescue was observed best with Igf1 and decreased gradually in treatments with iloprost, cPFT and Z-DEVD-FMK which is correlated with the nature of the compounds acting at more downstream positions of the PCD pathway. (B) TE integrity is maintained in expanded blastocysts derived from prolonged culture of homozygous EcNc ki/ki embryos and NcEc ki/ki embryos treated with Igf1. Embryos were isolated at E2.5 and observed for 24 h for blastocyst formation. After removal of zona pellucida and transfer to fresh medium embryos were cultured for additional 24 h to detect expansion. Embryos formed a properly expanded blastocyst with the exception of untreated NcEc ki/ki and E-cad−/− embryos that did not show signs of cavity formation. (C) Prolonged in vitro culture of EcNc ki/ki embryos does not induce PCD. Untreated EcNc and NcEc homozygous embryos were stained for active Caspase 3 after prolonged culture to monitor apoptosis in 3D reconstructed confocal images. Whereas many active Caspase 3-positive cells were detected in NcEc ki/ki embryos, no increase in apoptotic cells are present in EcNc ki/ki. (D) Upon treatment of homozygous mutants by either iloprost, cPFT or Igf1 between E2.5 and E3.5, blastocyst formation was accomplished and active Caspase 3 immunoreactivity (red) decrease [file pgen.1002609.s004.tif]

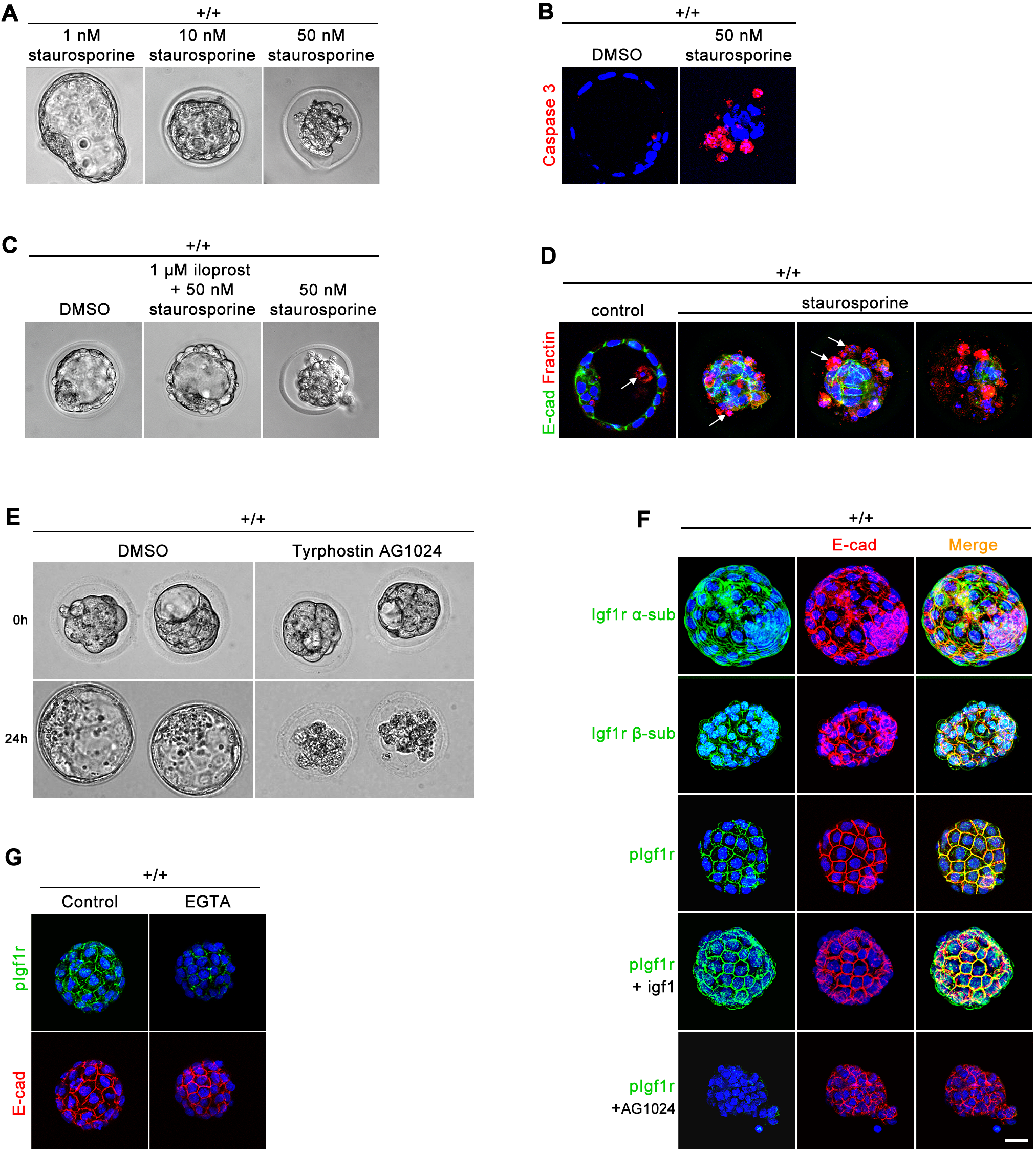

Supplement: Figure S5 — Staurosoporine and Tyrphostin AG1024 treatment and additional Igf1r expression analysis in wt embryos. (A) Staurosporine is a chemical compound that is inducing cleavage and activation of Caspase 3 and in turn leads to PCD. Wildtype embryos that have been incubated with 10 nM or 50 nM staurosporine were incapable of forming a proper blastocyst due to activation of Caspase 3. They underwent PCD within 24 h (middle and right), whereas 1 nM staurosporine had no effect (left). (B) 50 nM staurosporine treatment of wt embryos activated Caspase 3 as indicated by immunofluorescence labeling with an antibody recognizing the cleaved form of Caspase 3. (C) Pre-treatment of wt embryos with 1 µM iloprost for 24 h prevented staurosporine-induced apoptosis since iloprost is antagonizing the mode of activation of staurosporine. Double-treated embryos form a blastocyst similar to DMSO-treated controls. (D) Wildtype embryos treated by staurosporine show activation of Caspase 3 and phenocopied NcEc homozygous mutants. Upon Caspase 3 activation fragmented actin was detected by anti-Fractin labeling (red) and E-cad (green) was no longer detected in outside cells (arrows), whereas the ICM was more protected from the treatment. (E) Blocking Igf1r signaling prevents blastocyst formation in wt embryos. Embryos at E3.0 (top row) were treated with a specific inhibitor of Igf1r (Tyrphostin AG1024) and incubated for 24 h. Control embryos (left panel) form blastocoel cavities whereas treated littermates were incapable in forming a blastocyst, outside cells became fragmented and showed clear signs of apoptosis (right panel). (F) Distribution of total and active Igf1r shows partial overlap with E-cad. 3D reconstruction of z-stacks of same embryos as shown in Figure 5F and 5G. Labeling with antibodies detecting the α- and the β-subunit of Igf1r show localization of Igf1r throughout the membrane, partially overlapping with E-cad (first and second row, respectively). Overlap of the phosphorylated act [file pgen.1002609.s005.tif]
